# Supplementary material for: Synthesis of Novel 1,3,4-Oxadiazole-Derived α-Aminophosphonates/α-Aminophosphonic Acids and Evaluation of Their In Vitro Antiviral Activity against the Avian Coronavirus Infectious Bronchitis Virus
Source: Pharmaceutics. 2022 Dec 29;15(1):114. doi: 10.3390/pharmaceutics15010114 (PMC9867332; doi:10.3390/pharmaceutics15010114)
Supplement: Supplementary file 1 [file pharmaceutics-15-00114-s001.zip › pharmaceutics-2072001-supplementary.pdf]

File S1

Synthesis of novel 1,3,4-oxadiazole-derived α-aminophosphonates/α-aminophosphonic acids and evaluation of their in vitro antiviral activity against the avian coronavirus infectious bronchitis virus

Shaima Hkiri, Marwa Mekni-Toujani, Elvan Üstün, Karim Hosni, Abdeljelil Ghran, Soufiane Touil, Ali Samarat and David Sémeril

Contents

Characterising data of:

- 2-amino-5-phenyl-1,3,4-oxadiazole (**1**) p 3
- (*E*)-1-(4-trifluoromethylphenyl)-*N*-(5-phenyl-1,3,4-oxadiazol-2-yl)methanimine (**2a**) p 4
- (*E*)-1-(4-nitrophenyl)-*N*-(5-phenyl-1,3,4-oxadiazol-2-yl)methanimine (**2b**) p 6
- (*E*)-1-(4-fluorophenyl)-*N*-(5-phenyl-1,3,4-oxadiazol-2-yl)methanimine (**2c**) p 7
- (*E*)-1-(3,4,5-trifluophenyl)-*N*-(5-phenyl-1,3,4-oxadiazol-2-yl)methanimine (**2d**) p 9
- (*E*)-1-(3,5-bis-trifuoromethyl-phenyl)-*N*-(5-phenyl-1,3,4-oxadiazol-2-yl)methanimine (**2e**) p 11
- (*E*)-1-(4-trifluoromethoxyphenyl)-*N*-(5-phenyl-1,3,4-oxadiazol-2-yl)methanimine (**2f**) p 13
- dimethyl[(5-phenyl-1,3,4-axodiazol-2-ylamino)(4-trifluoromethylphenyl)methyl] phosphonate (**3**) p 15
- diphenyl[(5-phenyl-1,3,4-axodiazol-2-ylamino)(4-trifluoromethylphenyl)methyl] phosphonate (**4**) p 17
- diethyl[(5-phenyl-1,3,4-axodiazol-2-ylamino)(4-trifluoromethylphenyl)methyl] phospho-nate (**5a**) p 19
- diethyl[(5-phenyl-1,3,4-axodiazol-2-ylamino)(4-nitrophenyl)methyl]phosphonate (**5b**) p 21
- diethyl[(5-phenyl-1,3,4-axodiazol-2-ylamino)(4-fluorophenyl)methyl]phosphonate (**5c**) p 23
- diethyl[(5-phenyl-1,3,4-axodiazol-2-ylamino)(3,4,5-trifluophenyl)methyl]phosphonate (**5d**) p 25
- diethyl[(5-phenyl-1,3,4-axodiazol-2-ylamino)(3,5-bis-trifuoromethyl-phenyl)methyl] phosphonate (**5e**) p 27
- diethyl[(5-phenyl-1,3,4-axodiazol-2-ylamino)(4-trifluoromethoxyphenyl)methyl] phospho-nate (**5f**) p 29
- [(5-phenyl-1,3,4-axodiazol-2-ylamino)(4-trifluoromethylphenyl)methyl]phosphonic acid (**6a**) p 31
- [(5-phenyl-1,3,4-axodiazol-2-ylamino)(4-nitrophenyl)methyl]phosphonic acid (**6b**) p 33
- [(5-phenyl-1,3,4-axodiazol-2-ylamino)(4-fluorophenyl)methyl]phosphonic acid (**6c**) p 35

- [(5-phenyl-1,3,4-axodiazol-2-ylamino)(3,4,5-trifluophenyl)methyl]phosphonic acid (**6d**) p 37
- [(5-phenyl-1,3,4-axodiazol-2-ylamino)(3,5-bis-trifuoromethyl-phenyl)methyl] phosphonic acid (**6e**) p 39

X-ray structure of **5b** p 41

Interaction type and interaction residues of **5f** and **6a** with Papain-Like Protease (PDB ID: 4x2z) crystal structure p 43

Interaction type and interaction residues of **5f** and **6a** with N- and C-terminal domain of coronavirus nucleocapsid protein (PDB ID: 2c86) crystal structure p 43

Characterising data of 2-amino-5-phenyl-1,3,4-oxadiazole (1)

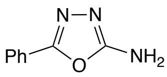

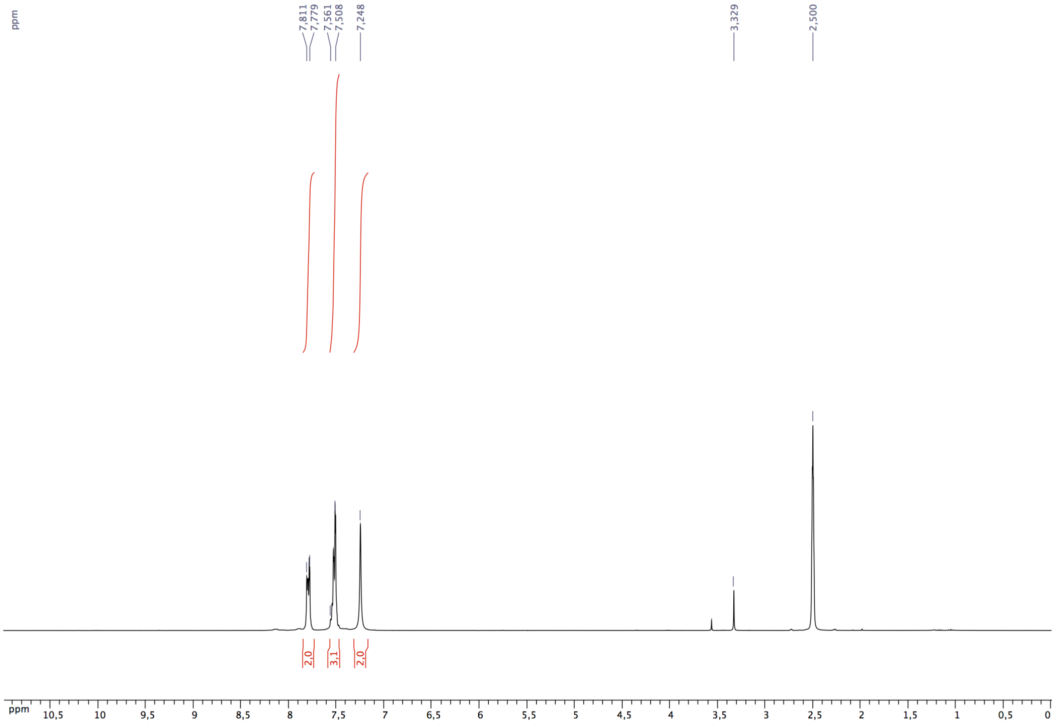

^{1}H NMR spectrum (DMSO-d_6)

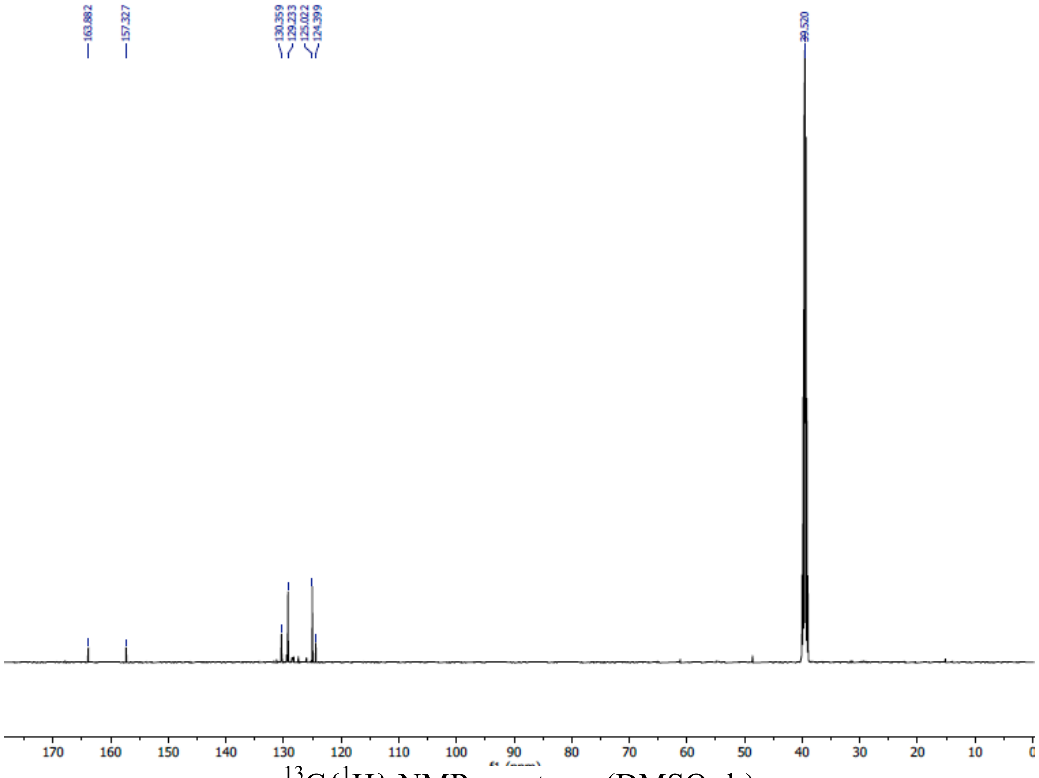

^{13}C{^{1}H} NMR spectrum (DMSO-d_6)

Characterising data of (*E*)-1-(4-trifluoromethylphenyl)-*N*-(5-phenyl-1,3,4-oxadiazol-2-yl)methanimine (2a)

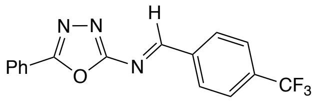

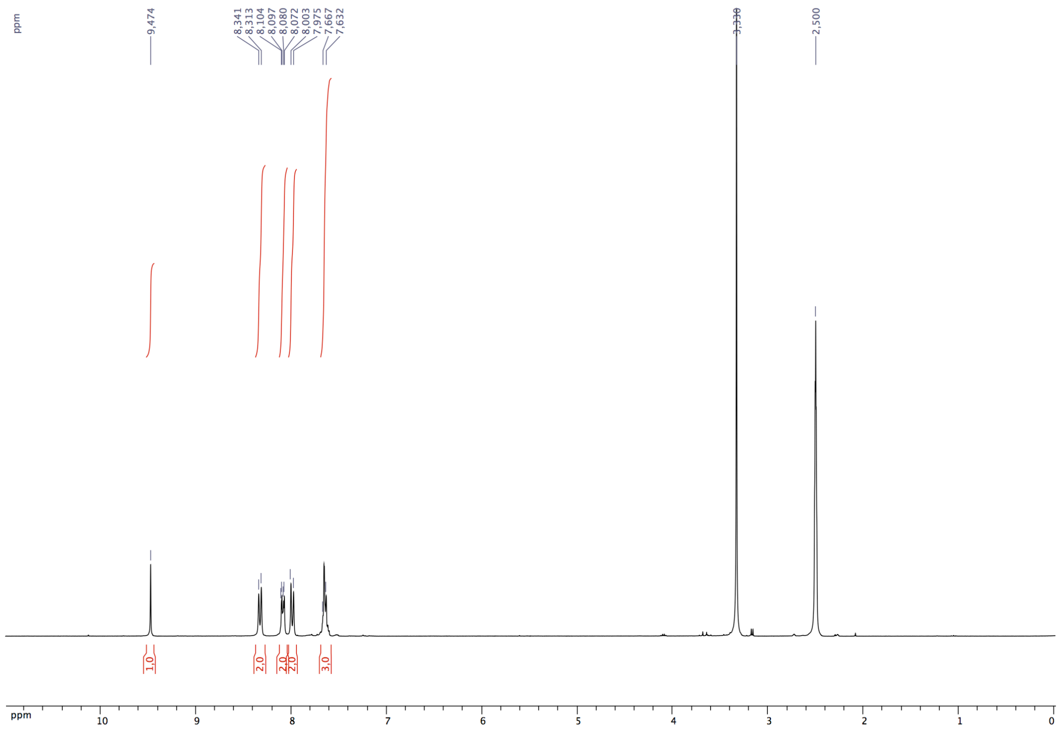

^{1}H NMR spectrum (DMSO-d_6)

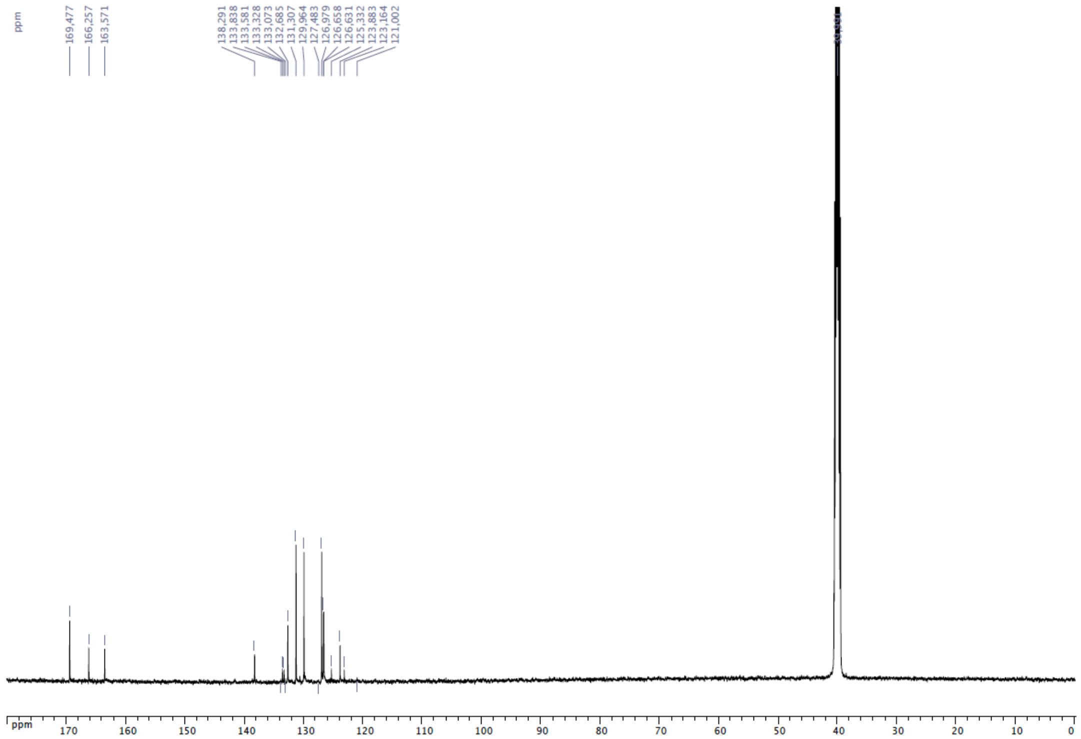

$^{13}C\{^1H\}$ NMR spectrum (DMSO-d_6)

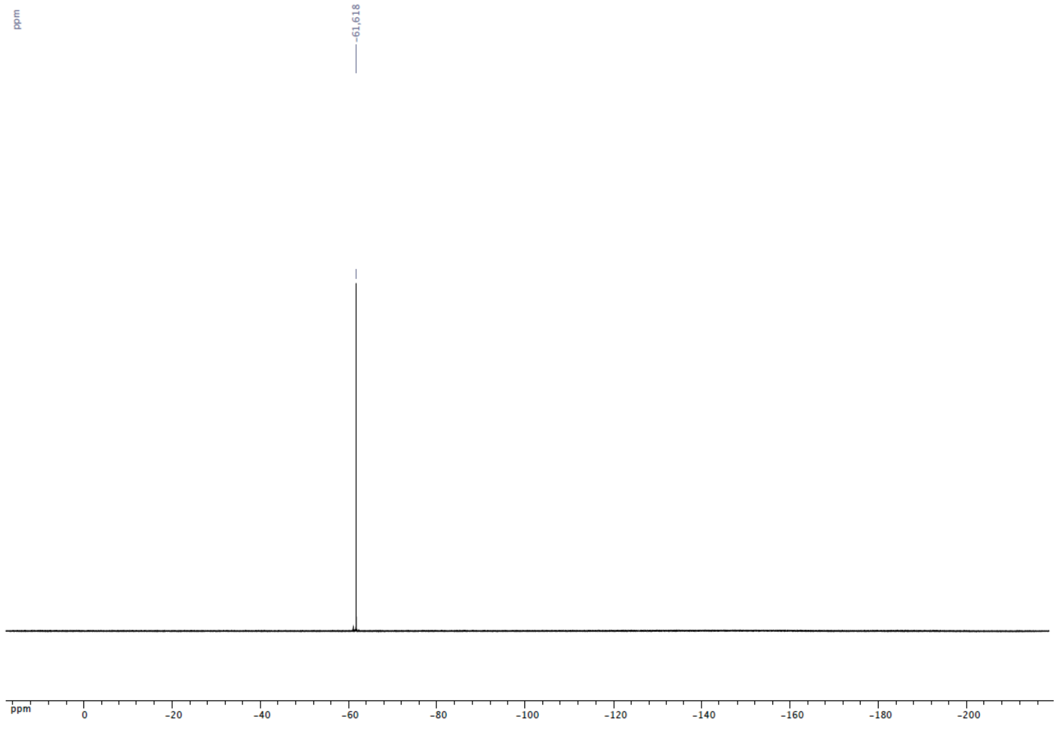

$^{19}F\{^1H\}$ NMR spectrum (DMSO-d_6)

Characterising data of (*E*)-1-(4-nitrophenyl)-*N*-(5-phenyl-1,3,4-oxadiazol-2-yl)methanimine (2b)

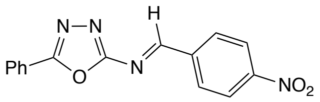

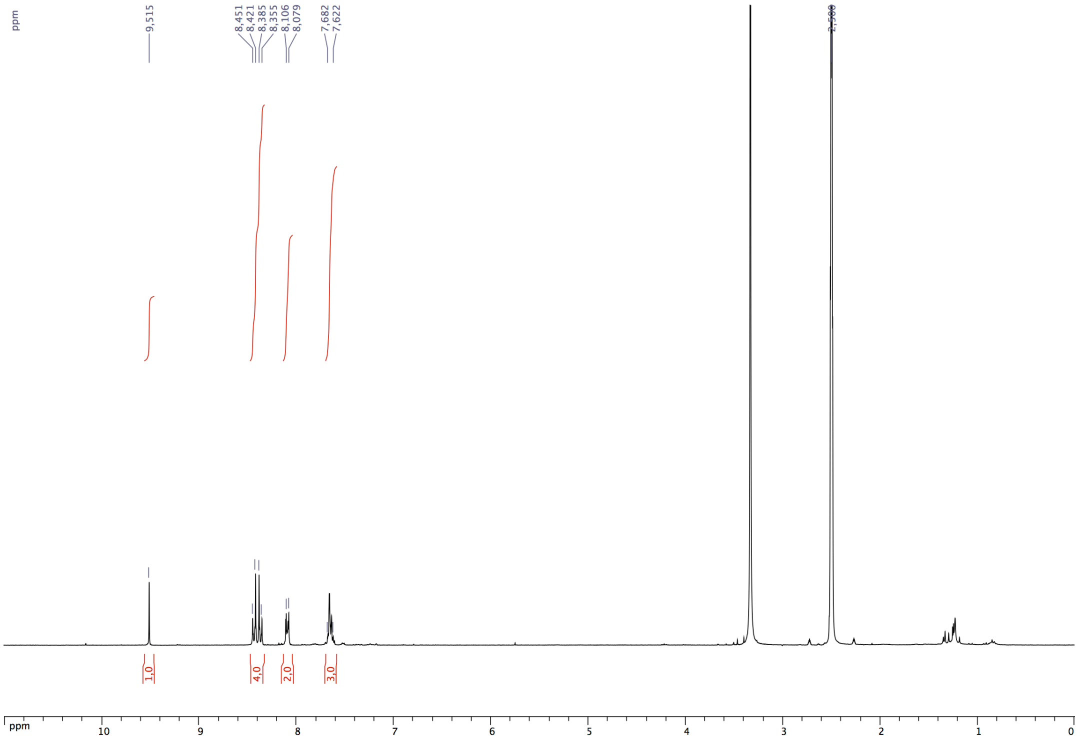

^{1}H NMR spectrum (DMSO-d_6)

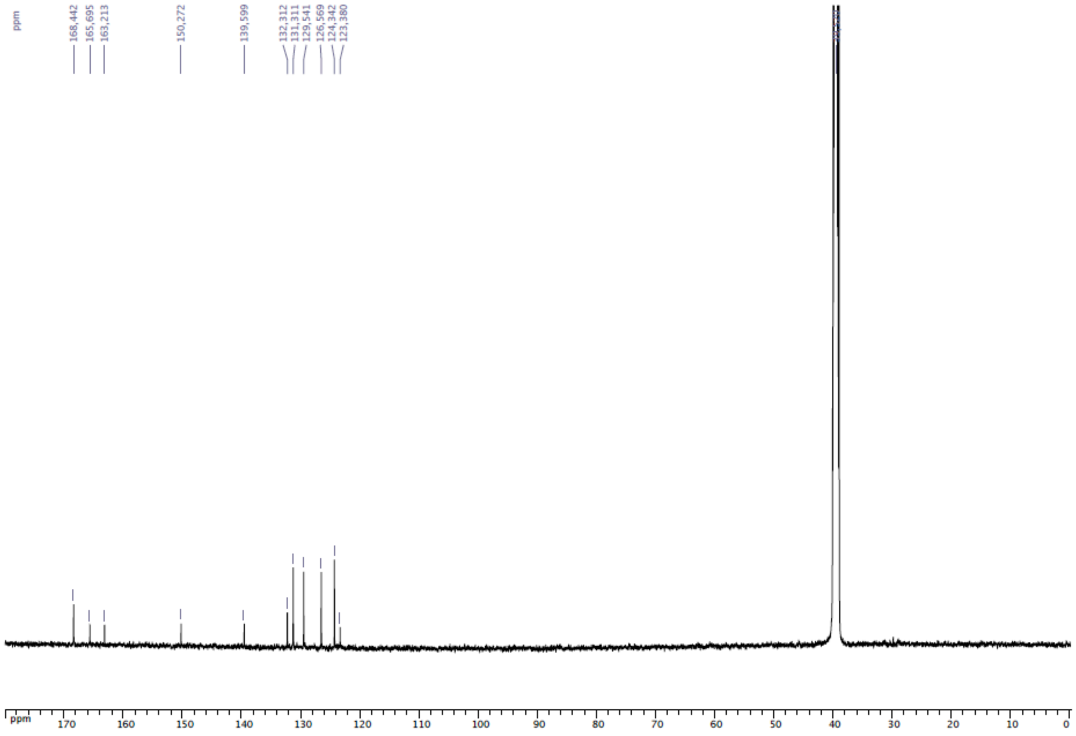

$^{13}C\{^{1}H\}$ NMR spectrum (DMSO-d_6)

Characterising data of (*E*)-1-(4-fuorophenyl)-
***N*-(5-phenyl-1,3,4-oxadiazol-2-yl)methanimine (2c)**

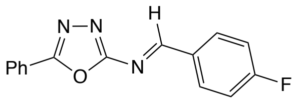

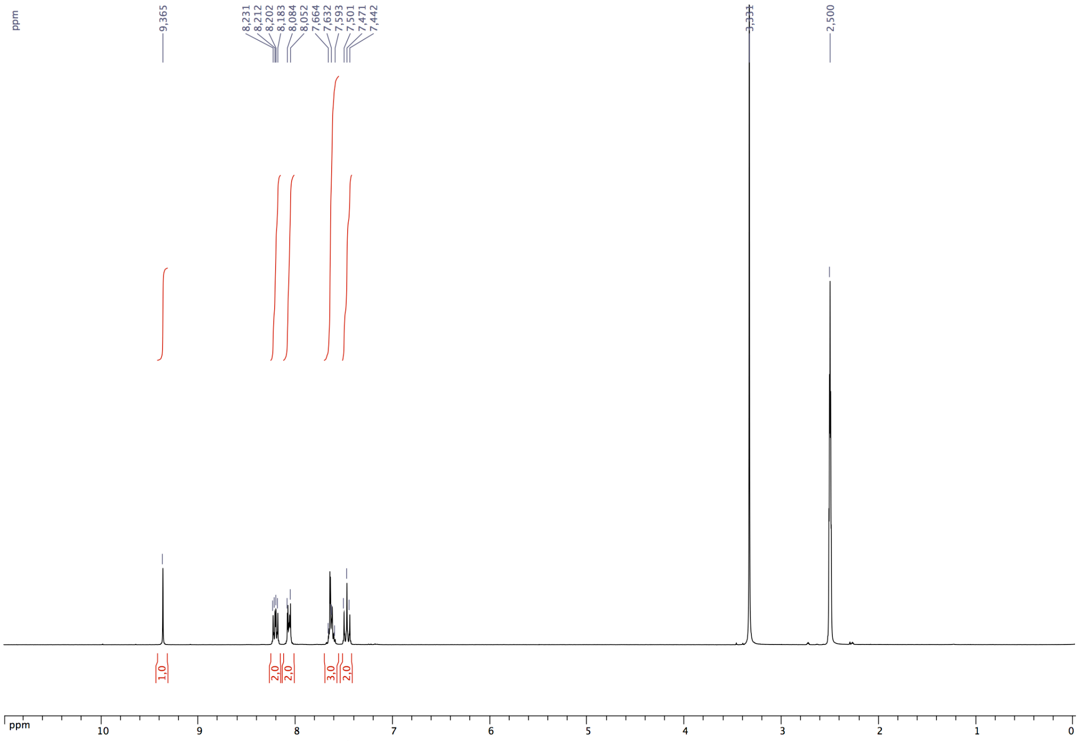

^{1}H NMR spectrum (DMSO-d_6)

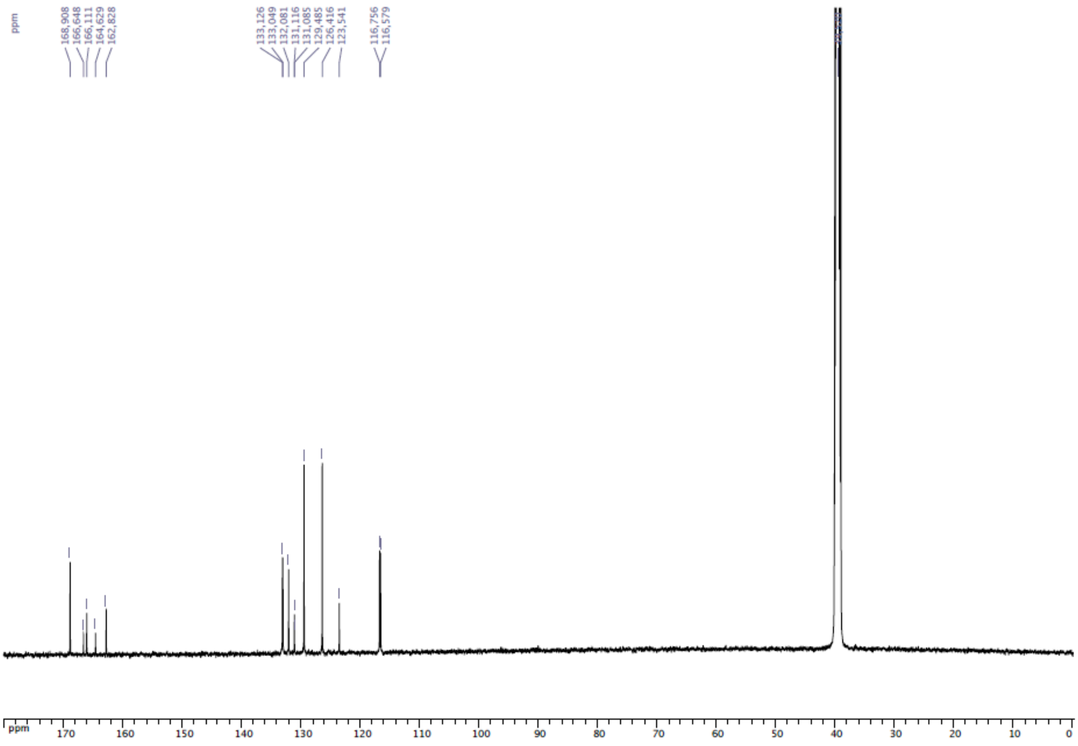

$^{13}C\{^{1}H\}$ NMR spectrum (DMSO-d_6)

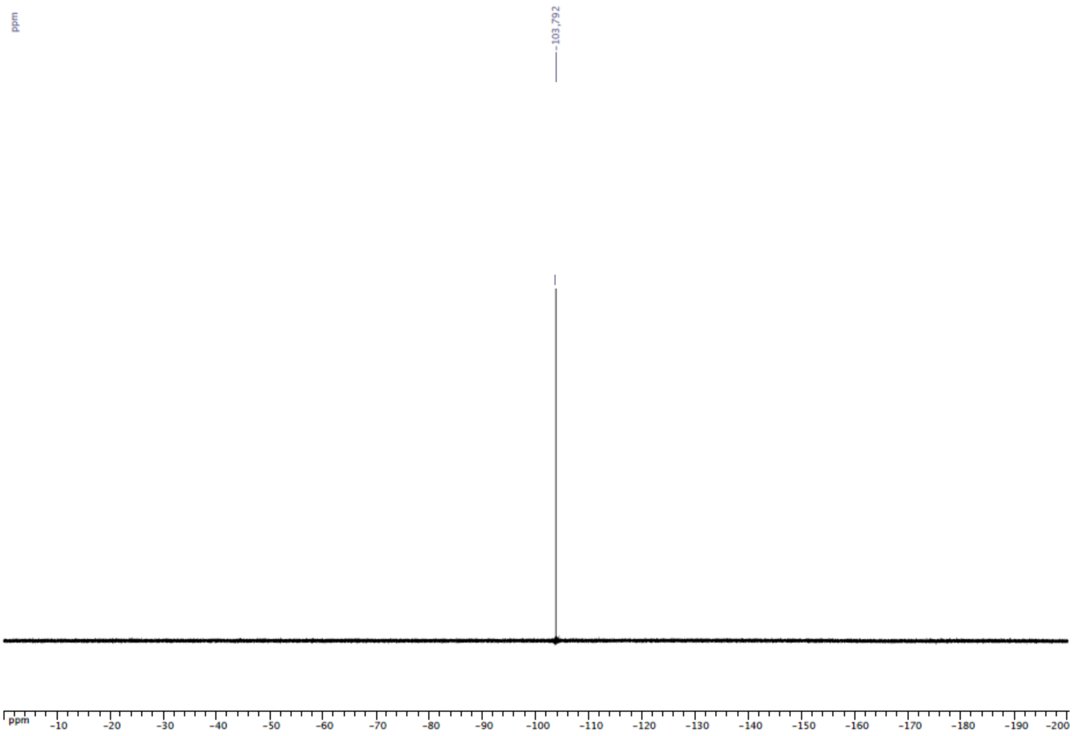

$^{19}F\{^{1}H\}$ NMR spectrum (DMSO-d_6)

Characterising data of (*E*)-1-(3,4,5-trifluophenyl)-*N*-(5-phenyl-1,3,4-oxadiazol-2-yl)methanimine (2d)

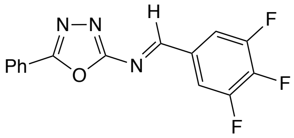

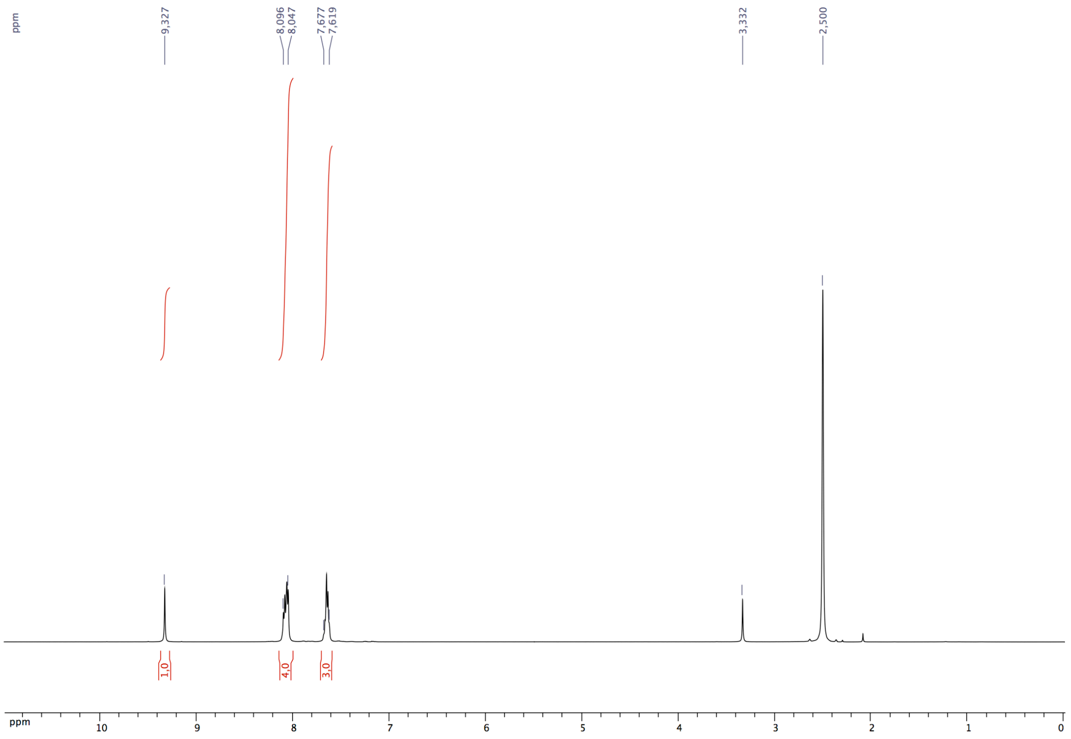

^{1}H NMR spectrum (DMSO-d$_6$)

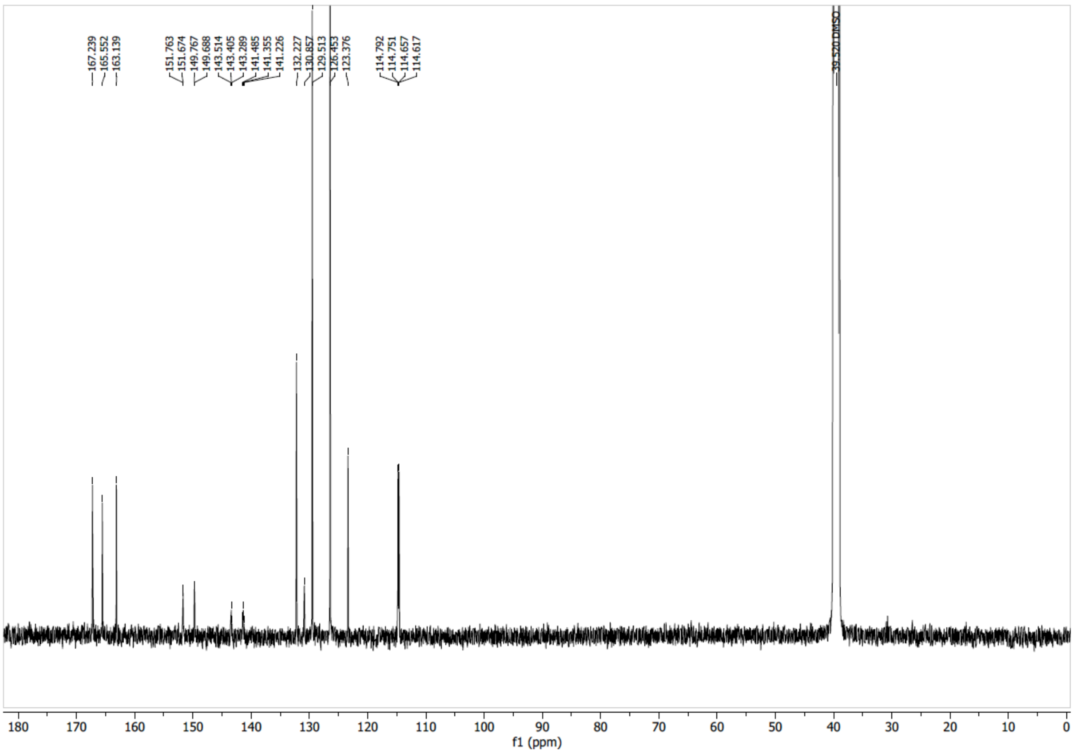

$^{13}C\{^1H\}$ NMR spectrum (DMSO-d_6)

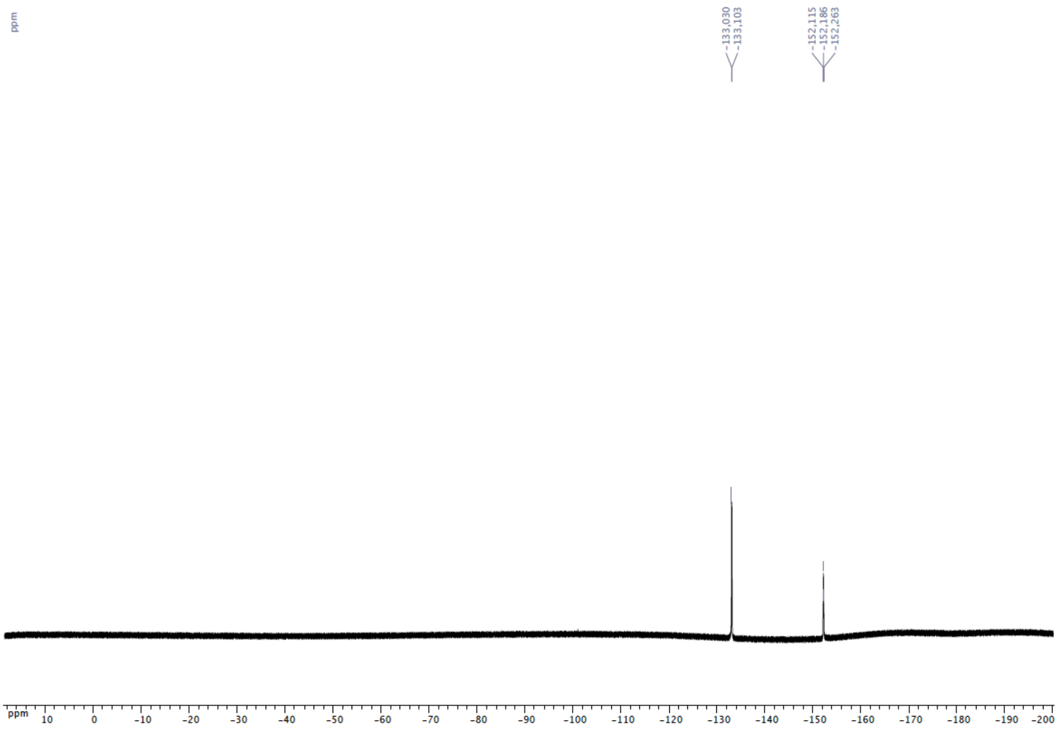

$^{19}F\{^1H\}$ NMR spectrum (DMSO-d_6)

Characterising data of (*E*)-1-(3,5-bis-trifuoromethyl-phenyl)-*N*-(5-phenyl-1,3,4-oxadiazol-2-yl)methanimine (2e)

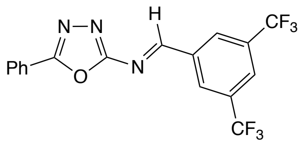

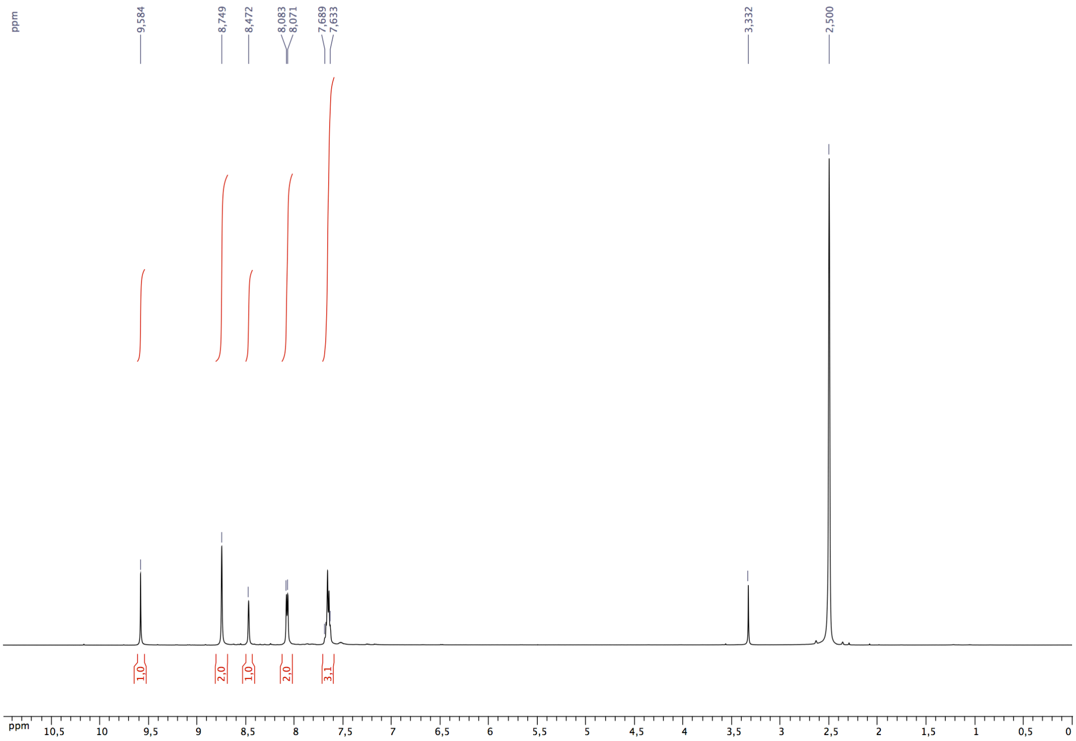

^{1}H NMR spectrum (DMSO-d$_6$)

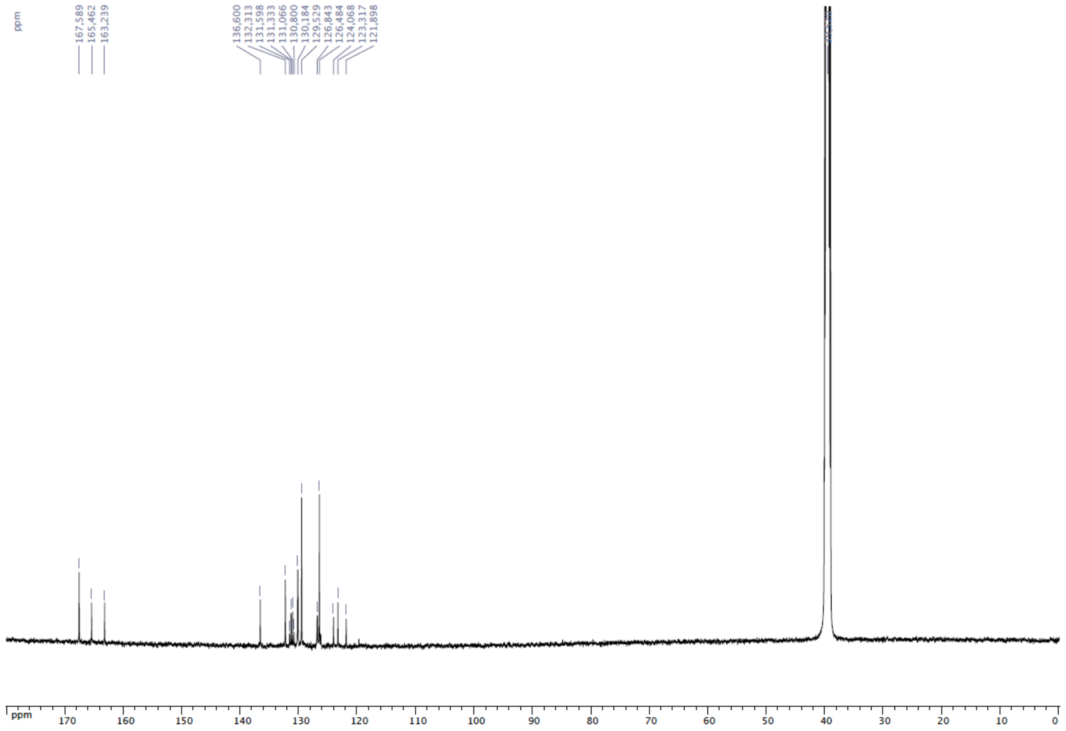

$^{13}C\{^{1}H\}$ NMR spectrum (DMSO-d_6)

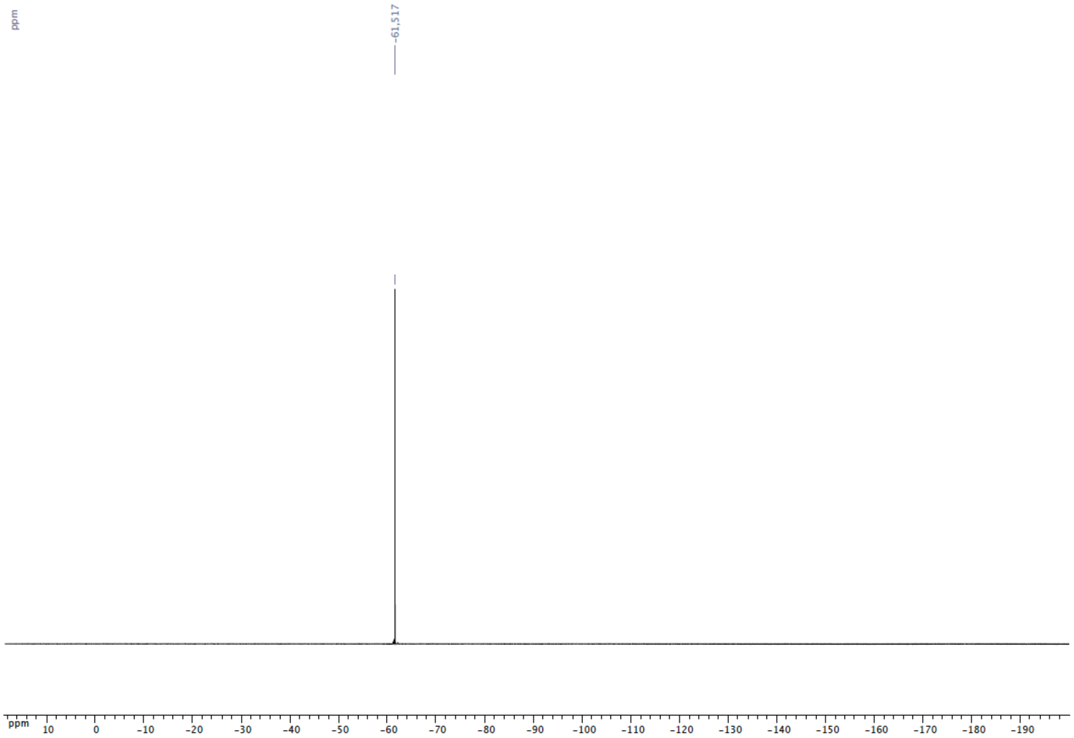

$^{19}F\{^{1}H\}$ NMR spectrum (DMSO-d_6)

Characterising data of (*E*)-1-(4-trifluoromethoxyphenyl)-*N*-(5-phenyl-1,3,4-oxadiazol-2-yl)methanimine (2f)

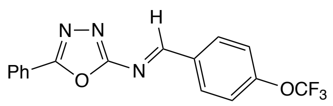

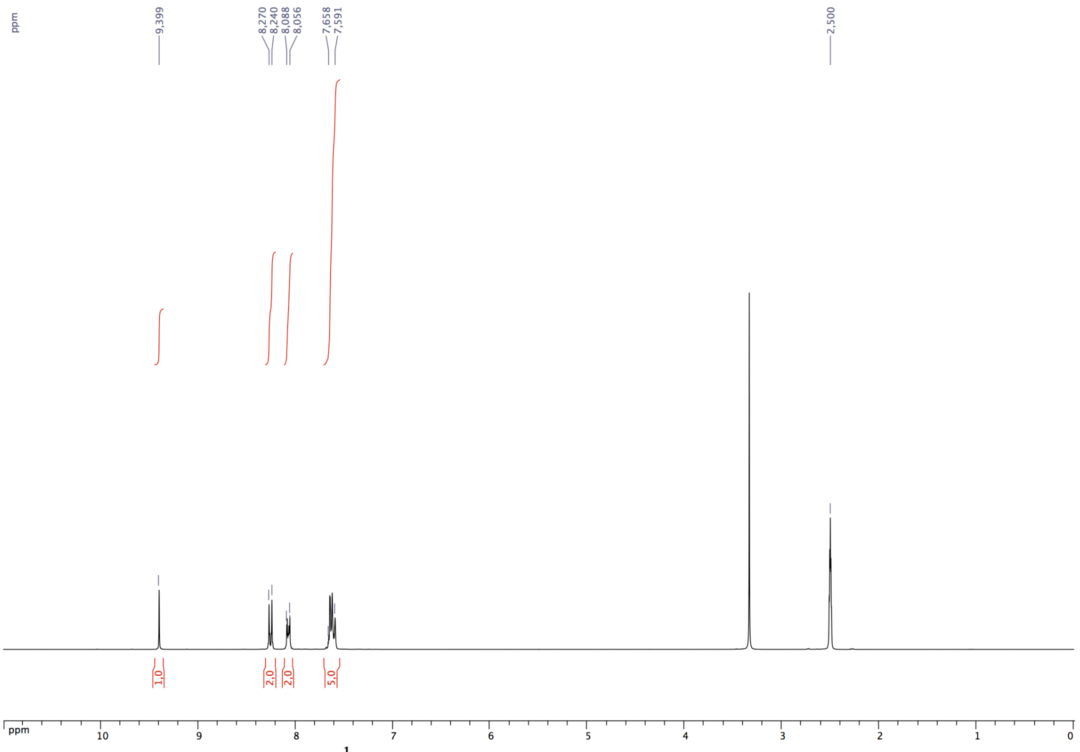

^{1}H NMR spectrum (DMSO-d$_6$)

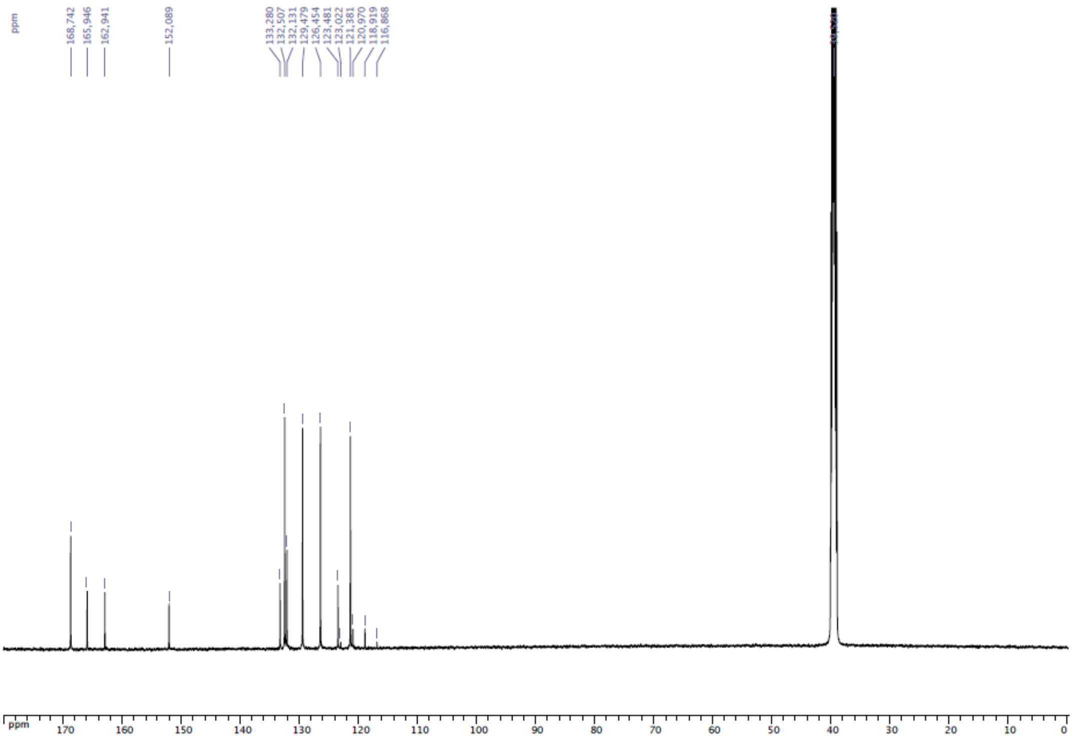

$^{13}C\{^{1}H\}$ NMR spectrum (DMSO-d_6)

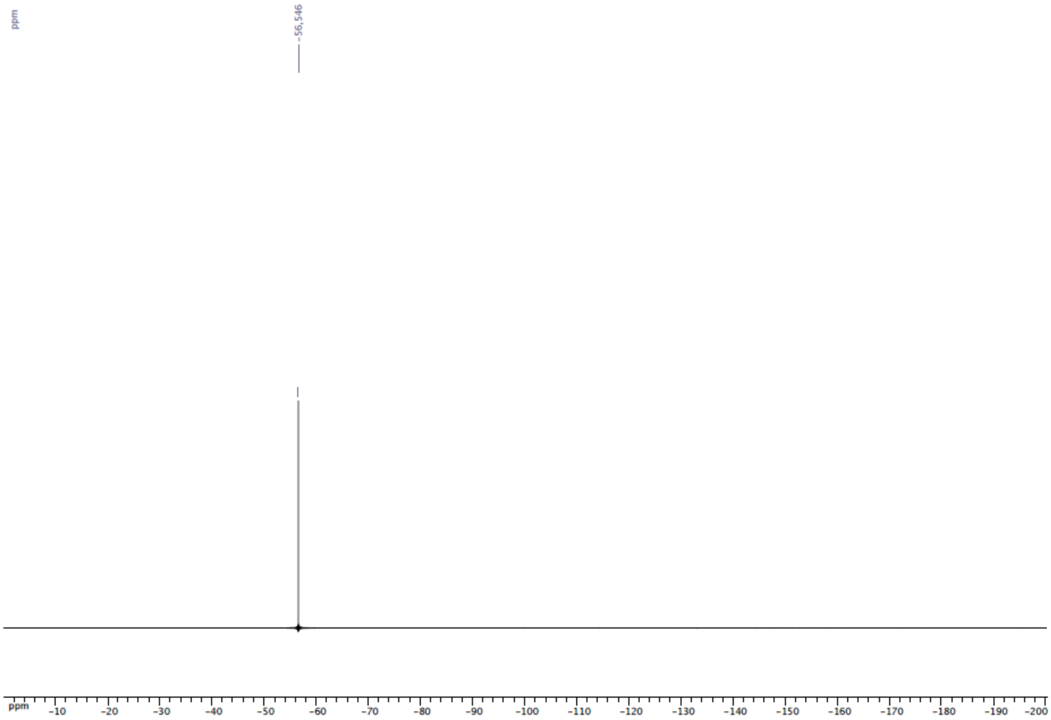

$^{19}F\{^{1}H\}$ NMR spectrum (DMSO-d_6)

Characterising data of dimethyl[(5-phenyl-1,3,4-axodiazol-2-ylamino)(4-trifluoromethylphenyl)methyl]phosphonate (3)

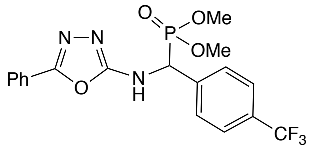

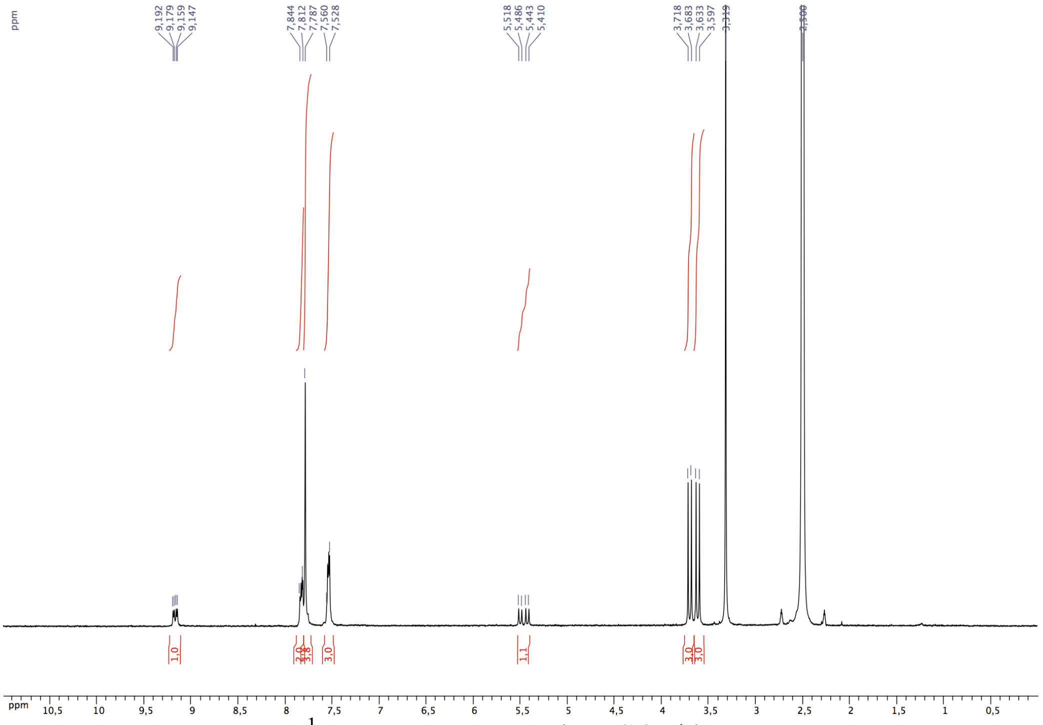

^{1}H NMR spectrum (DMSO-d$_6$)

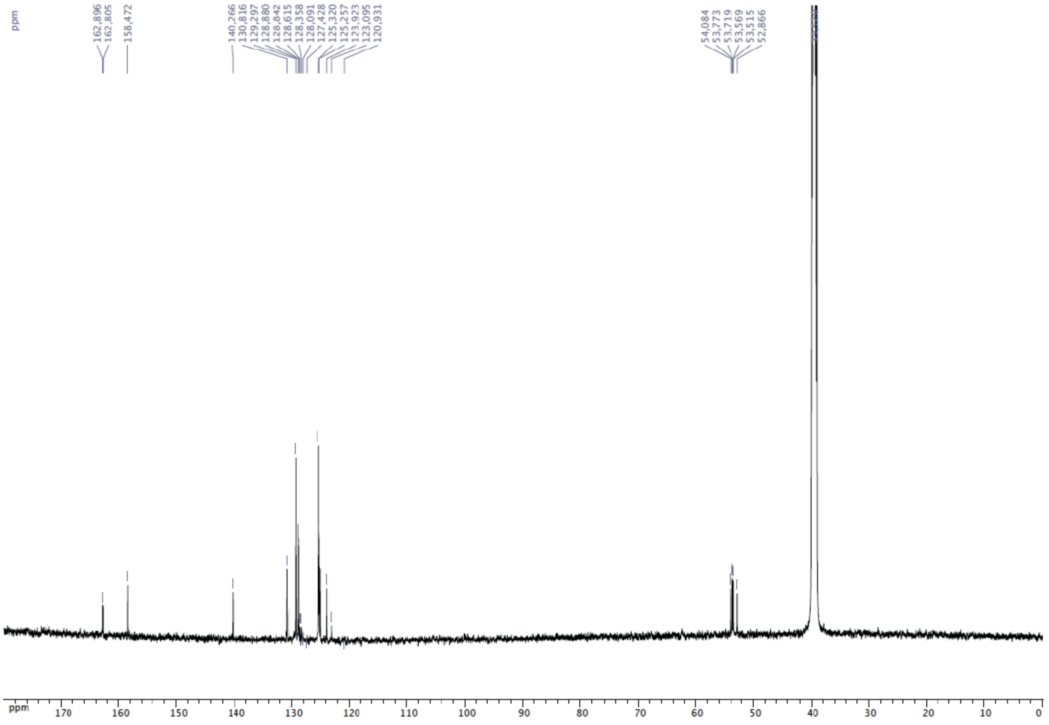

$^{13}C\{^{1}H\}$ NMR spectrum (DMSO-d$_6$)

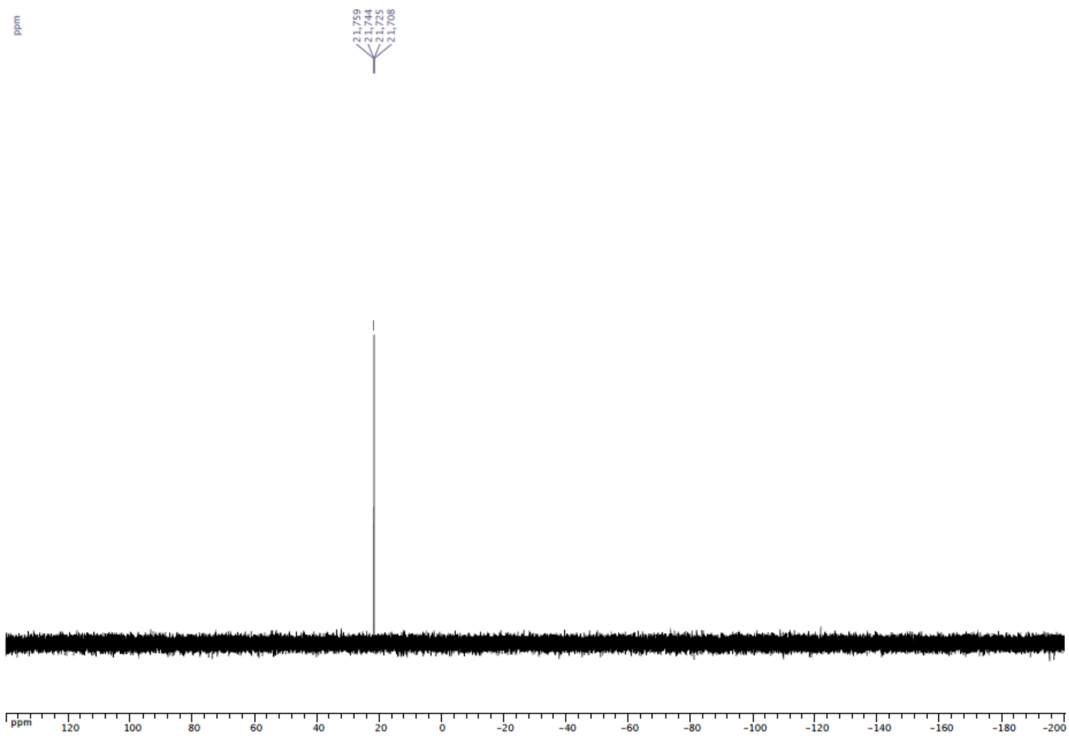

$^{31}P\{^{1}H\}$ NMR spectrum (DMSO-d_6)

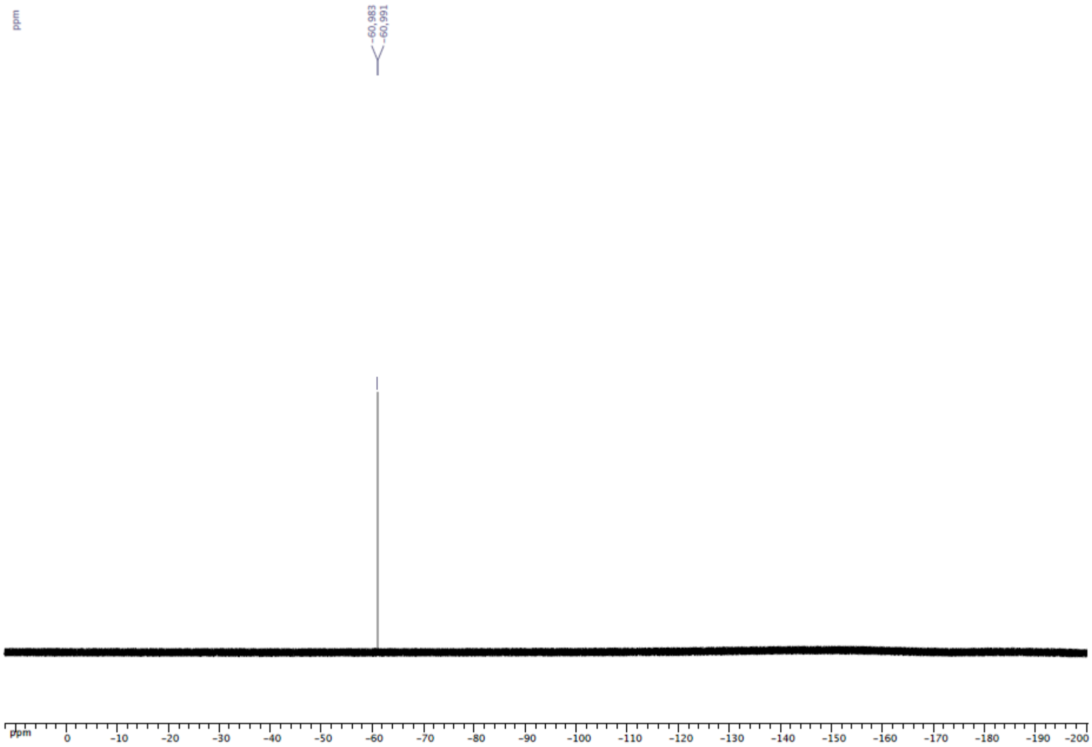

$^{19}F\{^{1}H\}$ NMR spectrum (DMSO-d_6)

Characterising data of diphenyl[(5-phenyl-1,3,4-axodiazol-2-ylamino) (4-trifluoromethylphenyl)methyl]phosphonate (4)

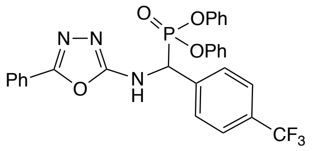

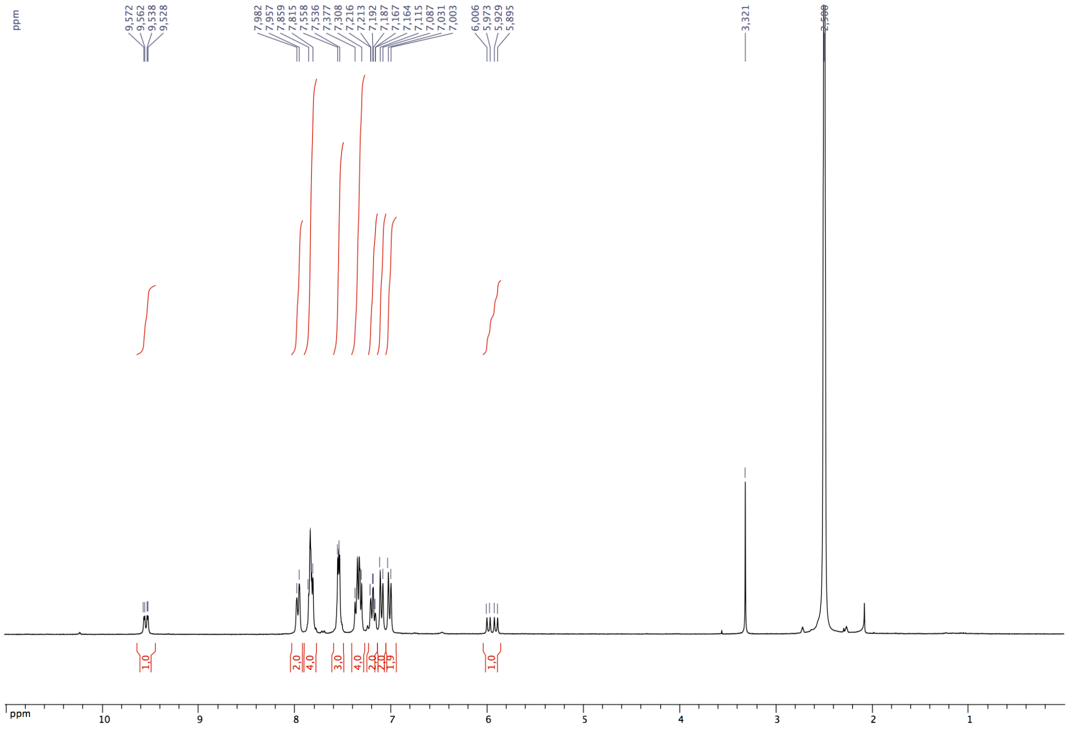

^{1}H NMR spectrum (DMSO-d$_6$)

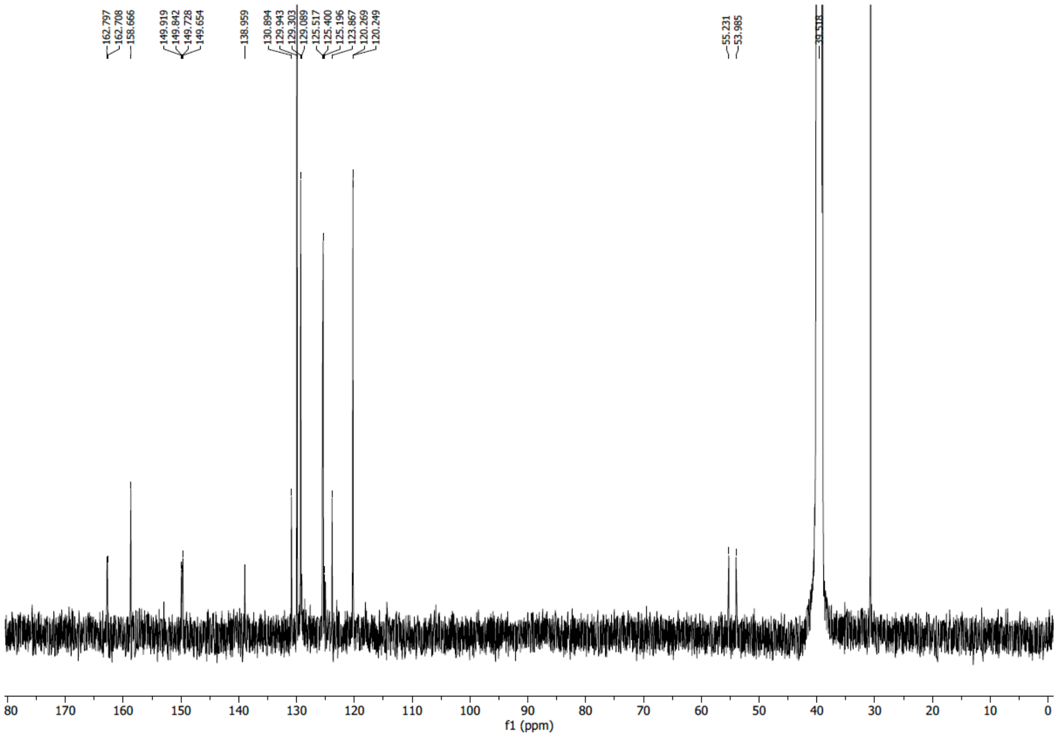

^{13}C{^{1}H} NMR spectrum (DMSO-d$_6$)

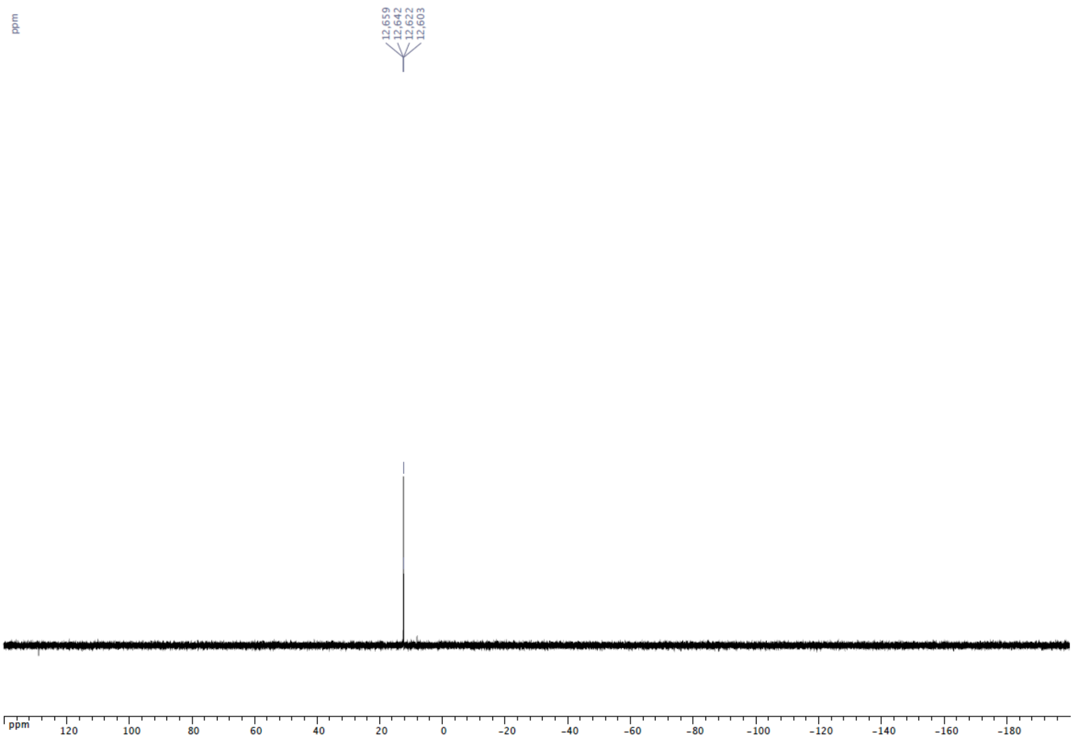

$^{31}P\{^{1}H\}$ NMR spectrum (DMSO-d_6)

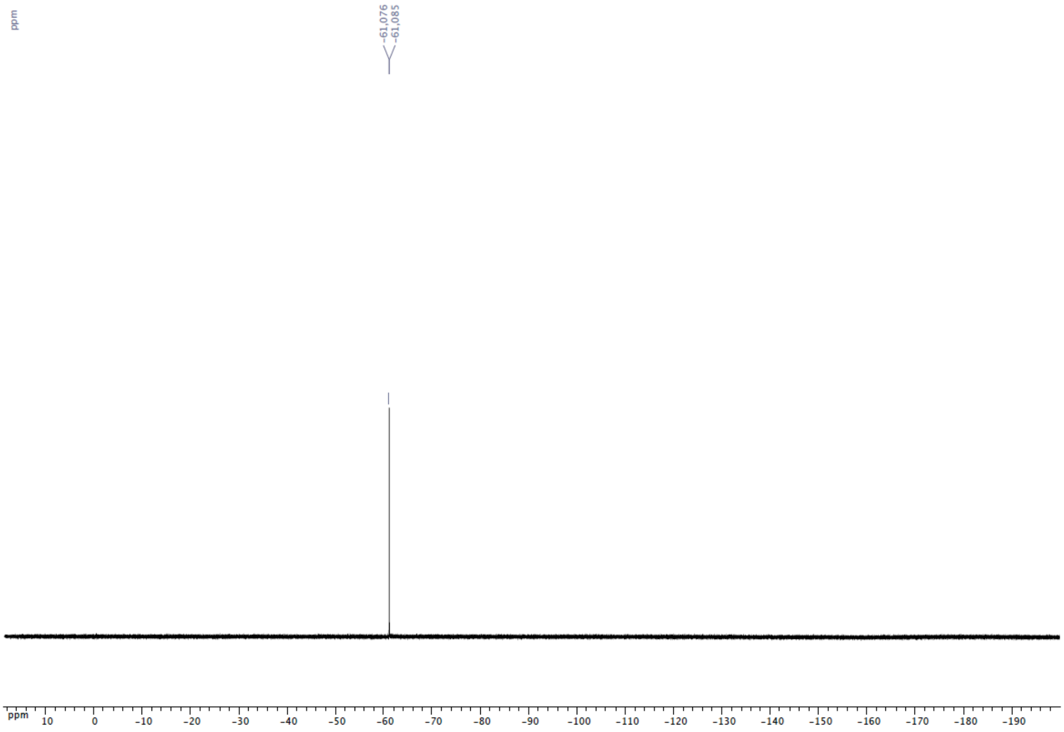

$^{19}F\{^{1}H\}$ NMR spectrum (DMSO-d_6)

Characterising data of diethyl[(5-phenyl-1,3,4-axodiazol-2-ylamino)(4-trifluoromethylphenyl)methyl]phosphonate (5a)

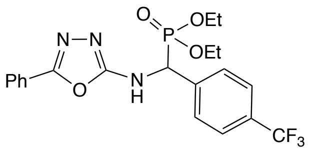

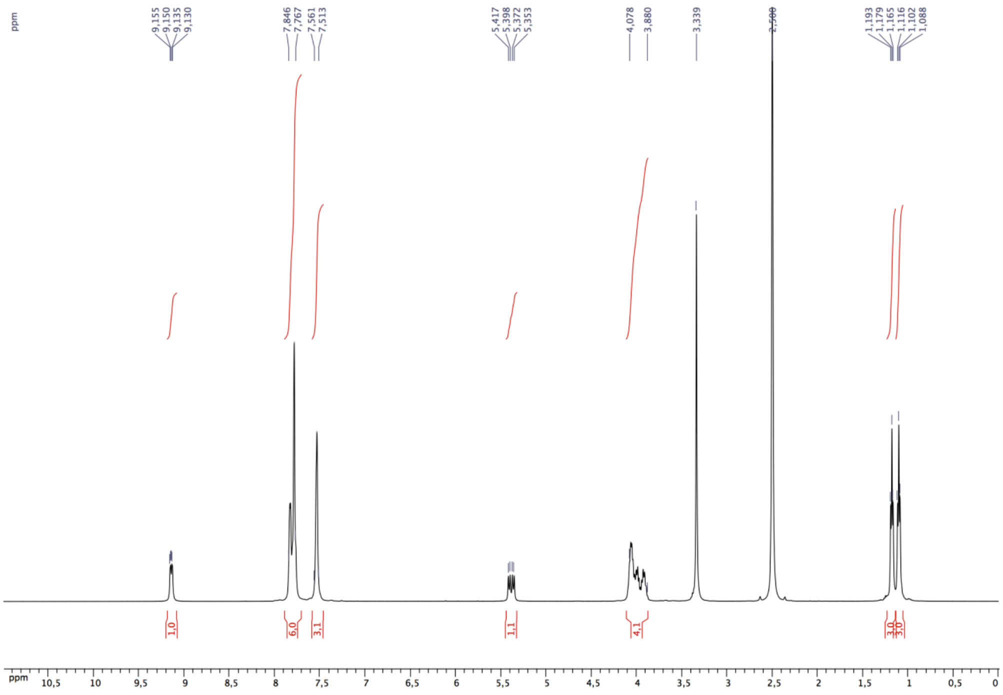

^{1}H NMR spectrum (DMSO-d$_6$)

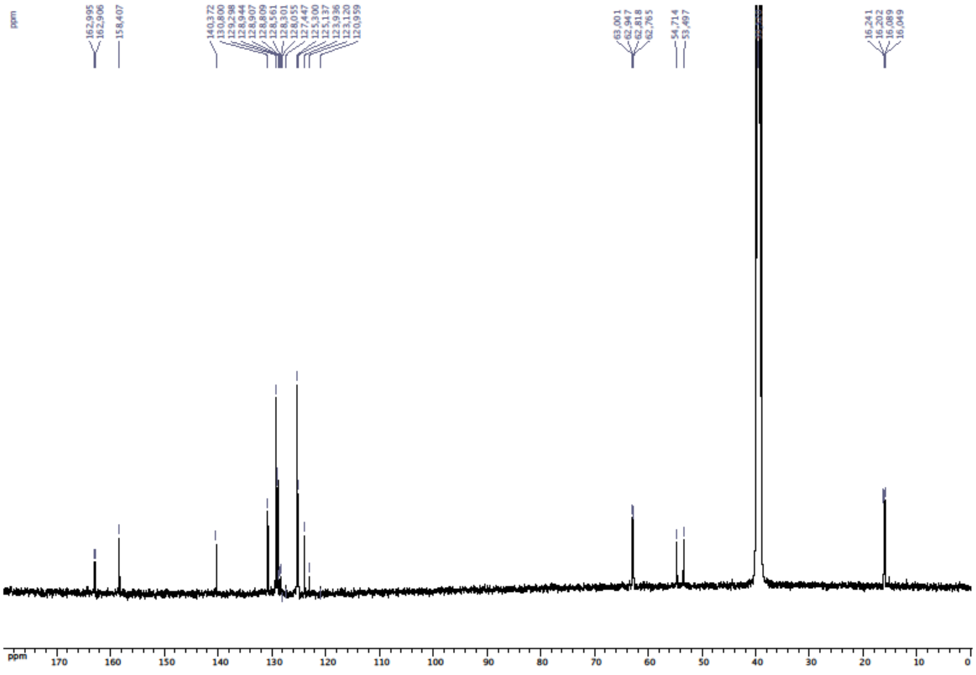

^{13}C{^{1}H} NMR spectrum (DMSO-d$_6$)

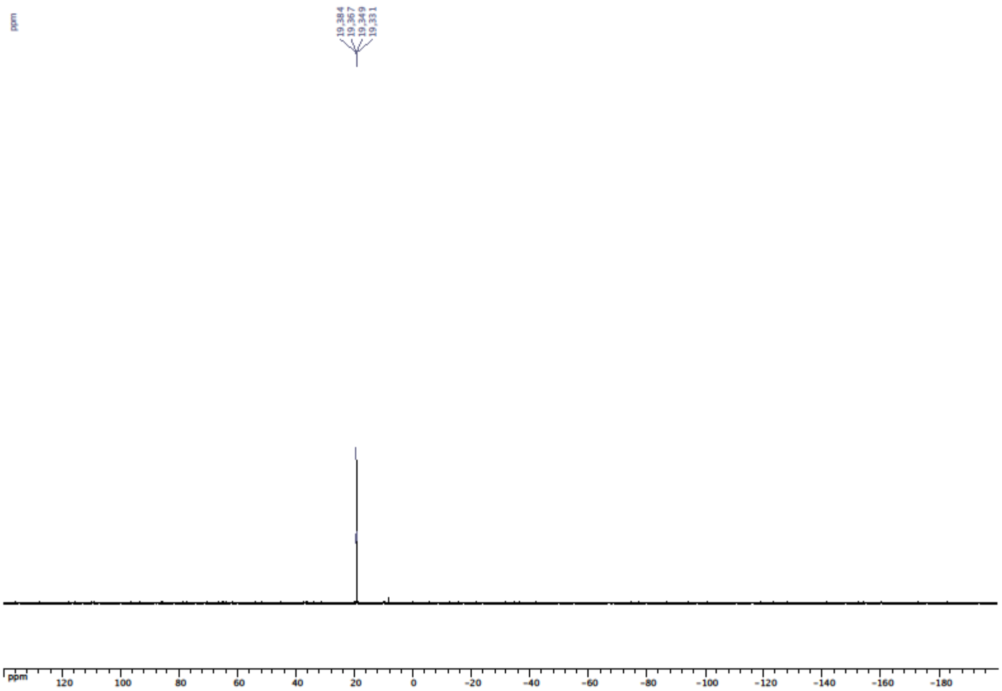

$^{31}P\{^{1}H\}$ NMR spectrum (DMSO-d_6)

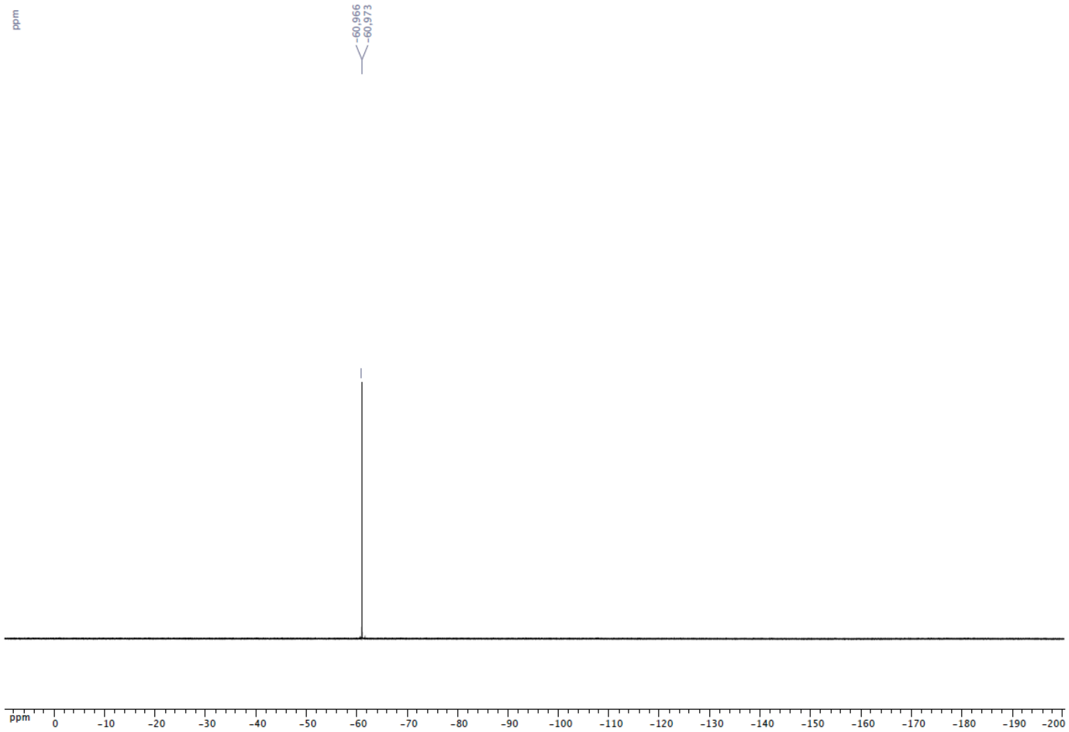

$^{19}F\{^{1}H\}$ NMR spectrum (DMSO-d_6)

Characterising data of diethyl[(5-phenyl-1,3,4-axodiazol-2-ylamino)(4-nitrophenyl)methyl]phosphonate (5b)

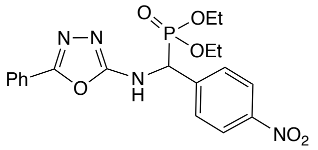

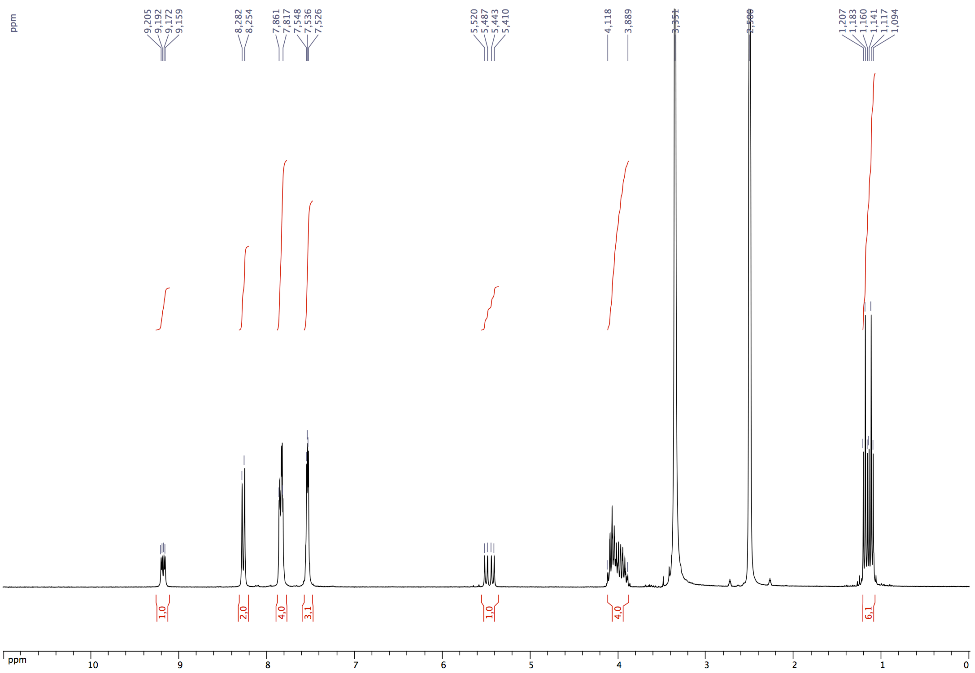

^{1}H NMR spectrum (DMSO-d_6)

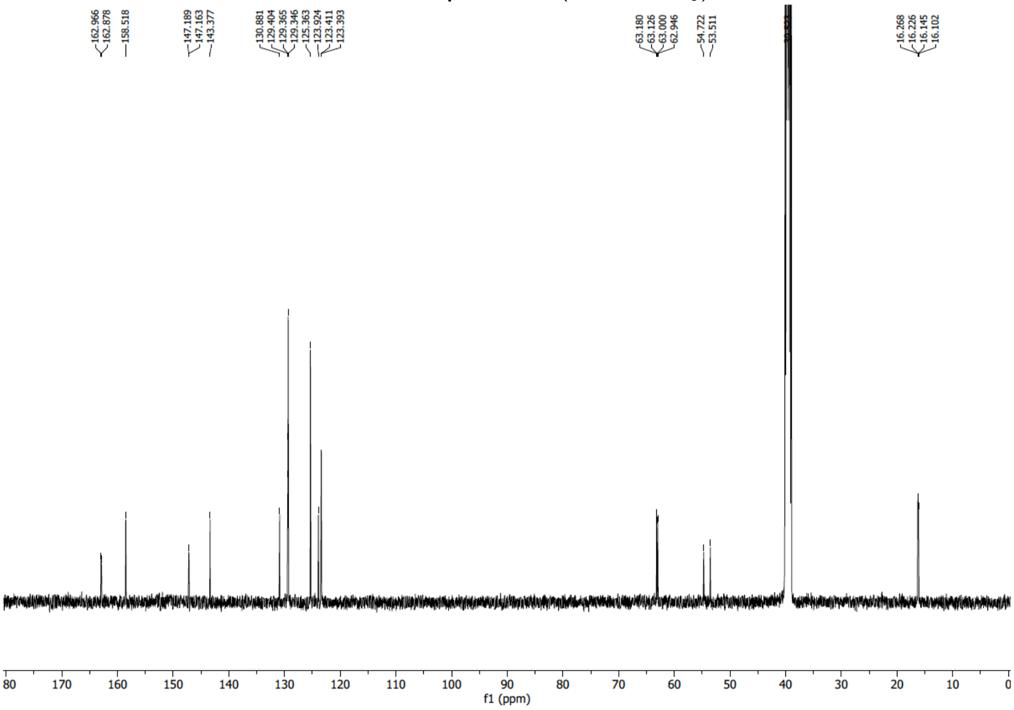

$^{13}C\{^{1}H\}$ NMR spectrum (DMSO-d_6)

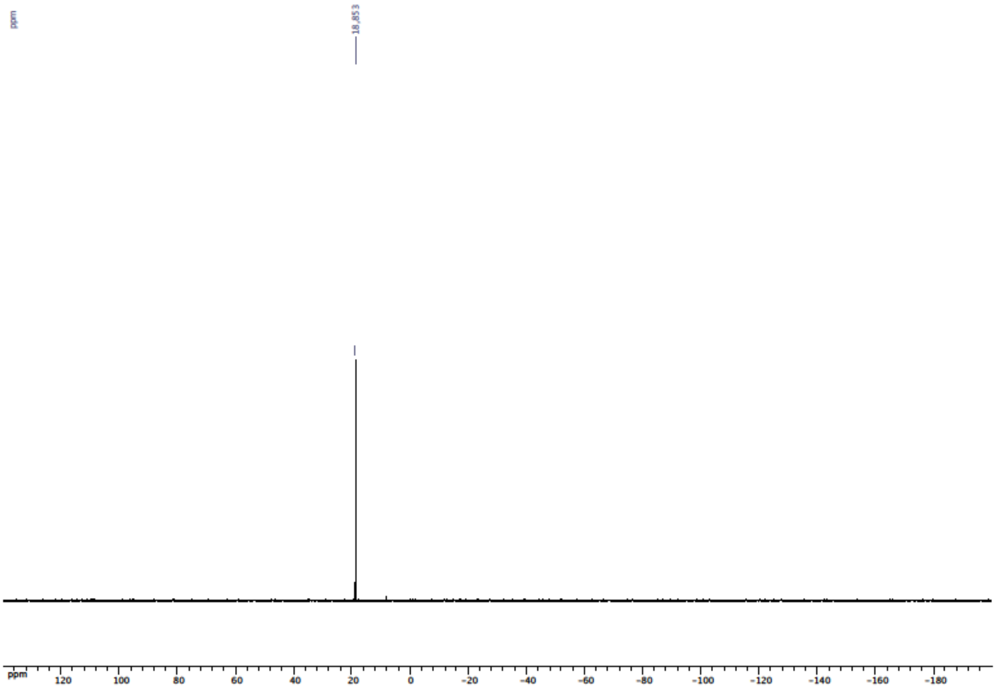

$^{31}P\{^{1}H\}$ NMR spectrum (DMSO-d_6)

Characterising data of diethyl[(5-phenyl-1,3,4-axodiazol-2-ylamino)(4-fluorophenyl)methyl]phosphonate (5c)

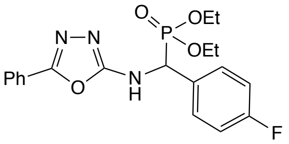

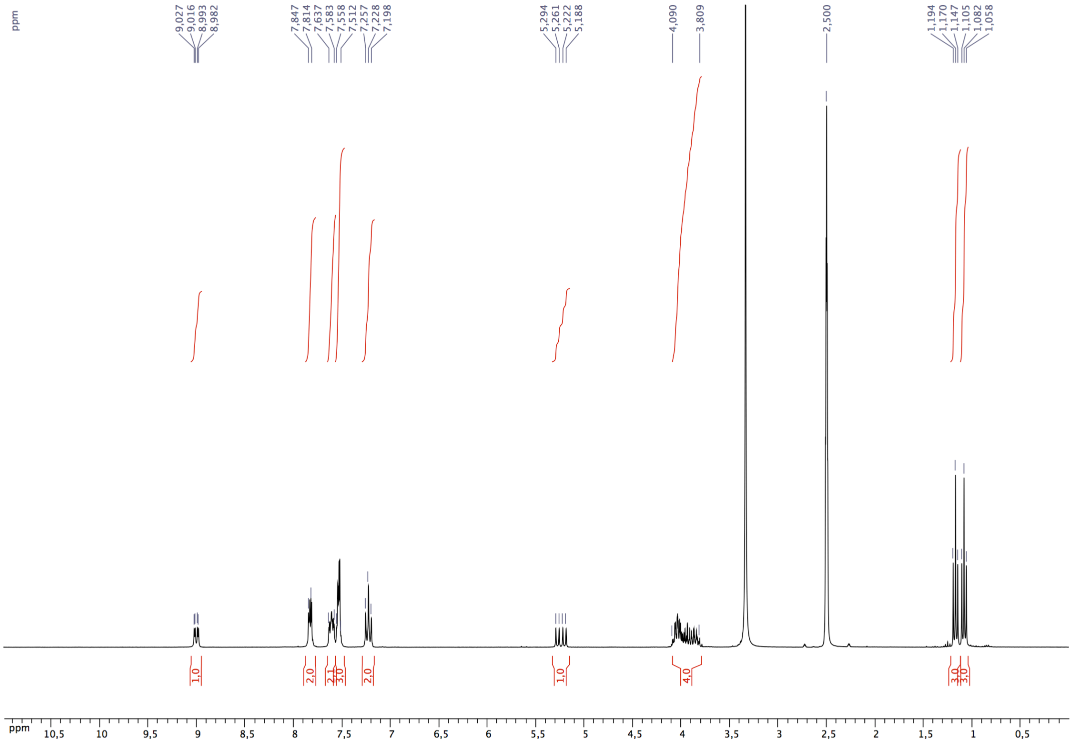

^1H NMR spectrum (DMSO-d_6)

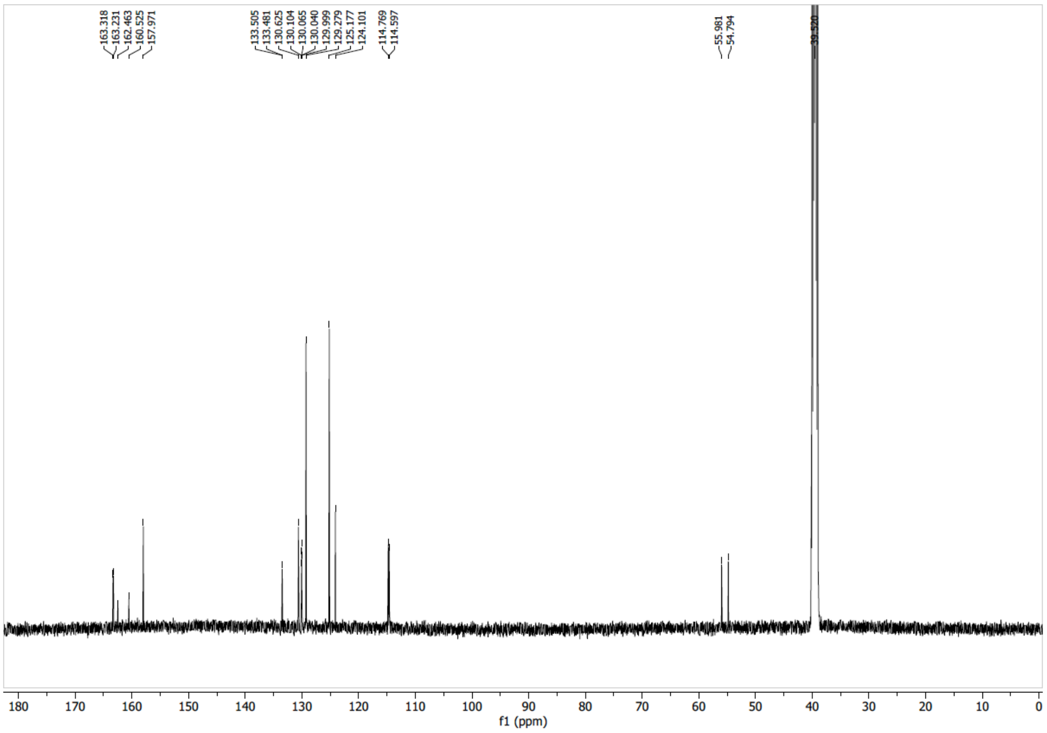

$^{13}C\{^1H\}$ NMR spectrum (DMSO-d_6)

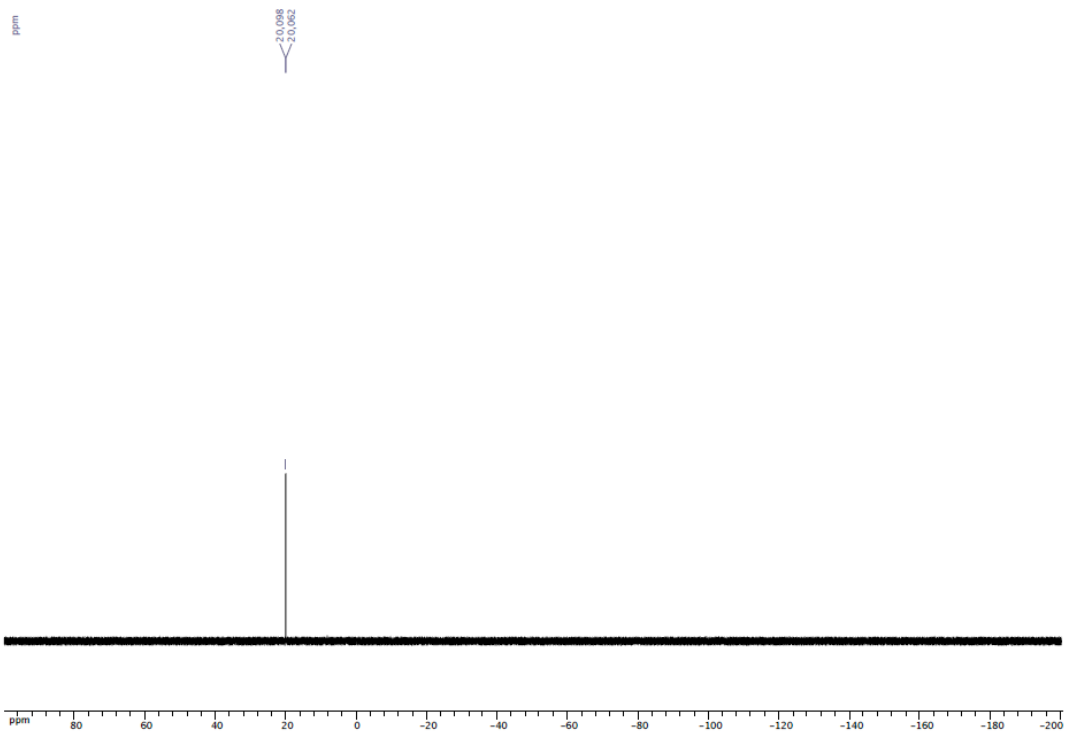

$^{31}P\{^{1}H\}$ NMR spectrum (DMSO-d_6)

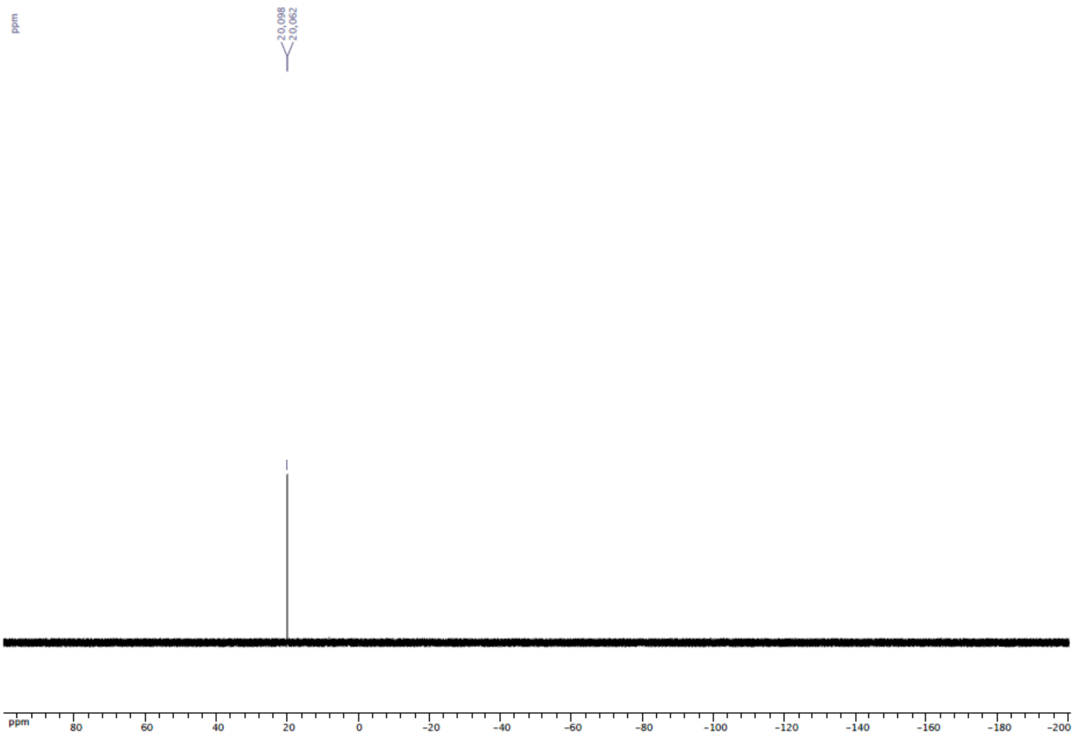

$^{19}F\{^{1}H\}$ NMR spectrum (DMSO-d_6)

Characterising data of diethyl[(5-phenyl-1,3,4-axodiazol-2-ylamino)(3,4,5-trifluophenyl)methyl]phosphonate (5d)

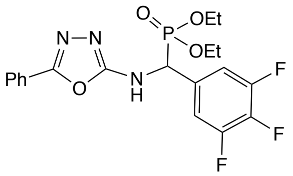

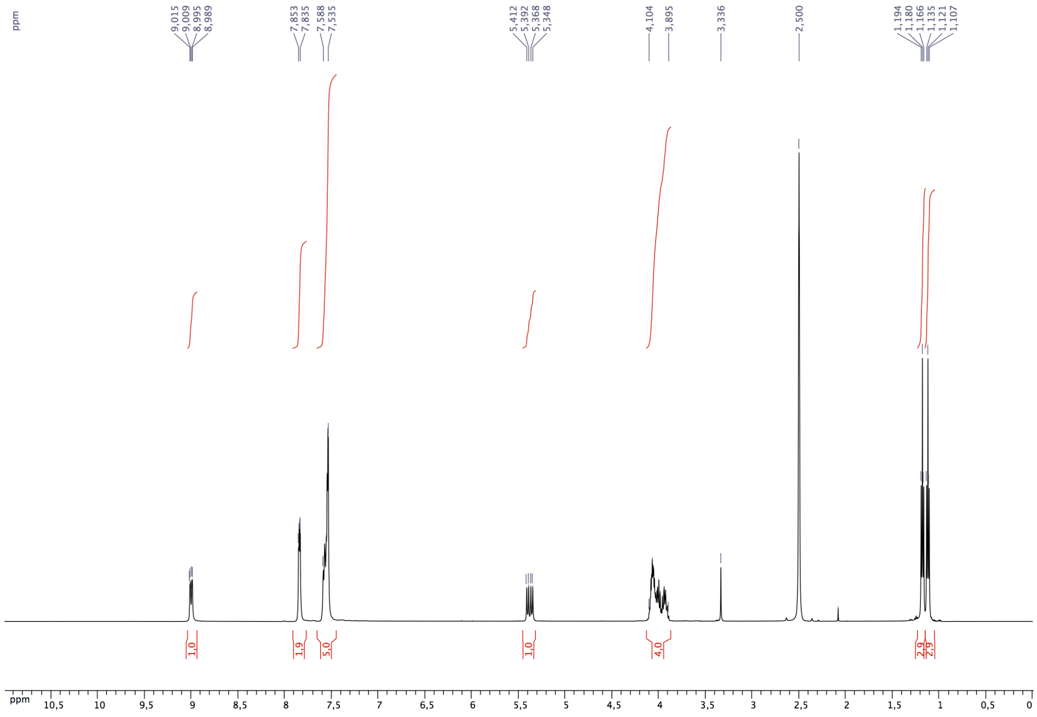

^{1}H NMR spectrum (DMSO-d$_6$)

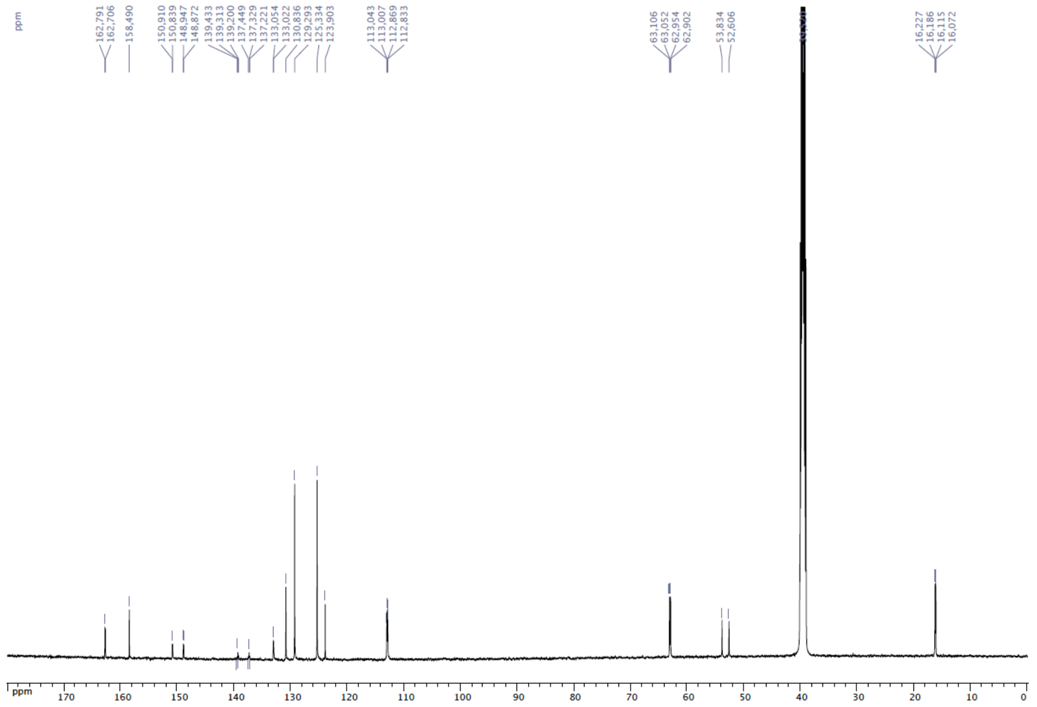

^{13}C{^{1}H} NMR spectrum (DMSO-d$_6$)

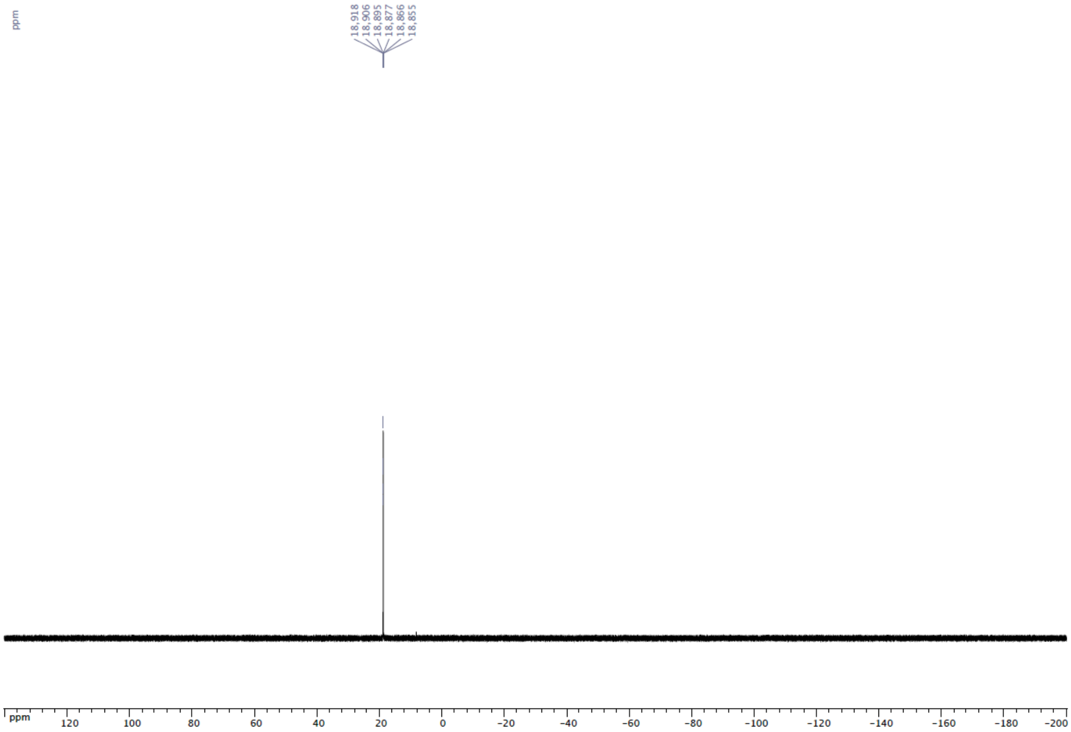

$^{31}P\{^{1}H\}$ NMR spectrum (DMSO-d_6)

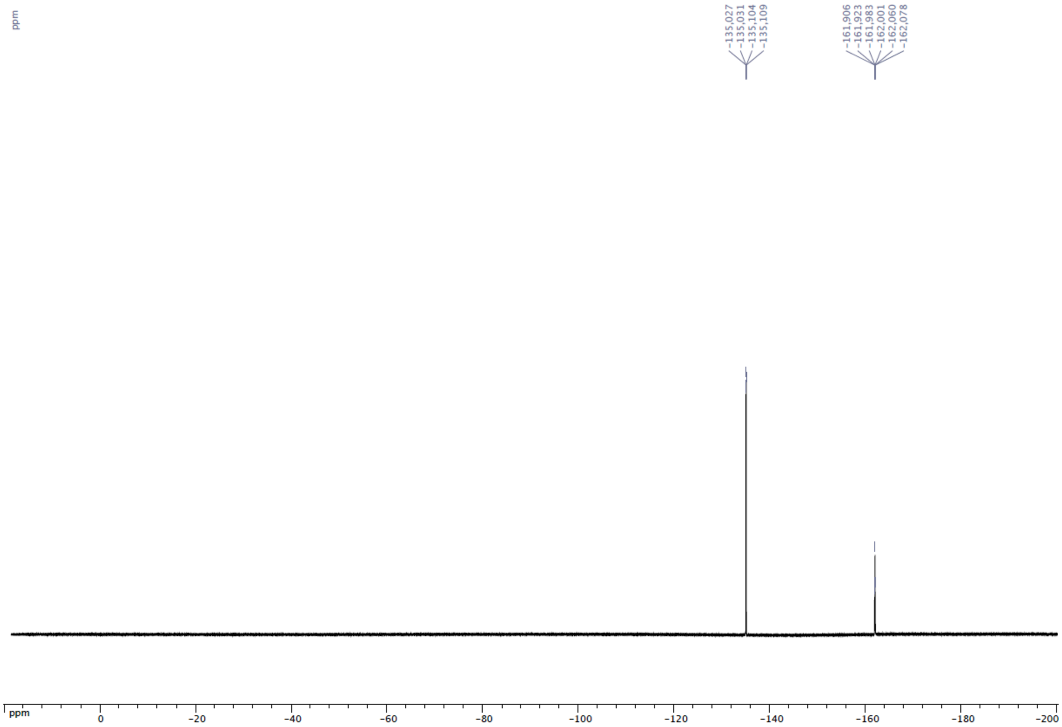

$^{19}F\{^{1}H\}$ NMR spectrum (DMSO-d_6)

Characterising data of diethyl[(5-phenyl-1,3,4-axodiazol-2-ylamino)(3,5-bis-trifuoromethyl-phenyl)methyl]phosphonate (5e)

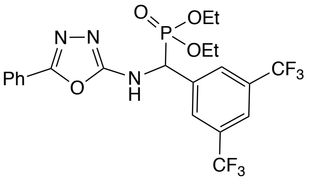

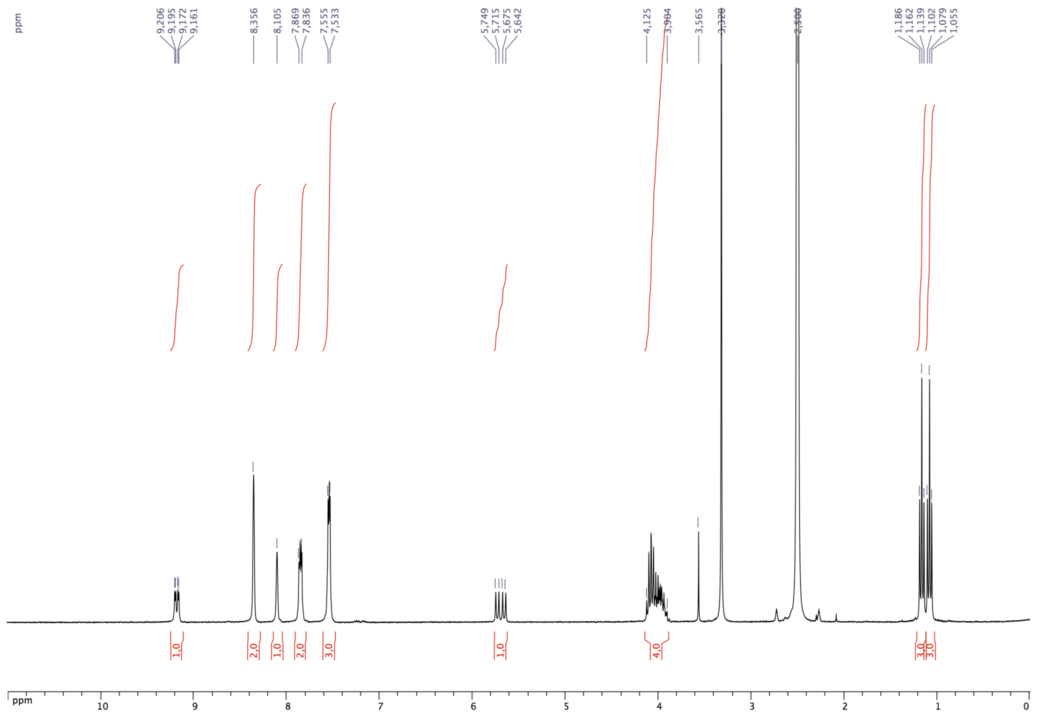

^{1}H NMR spectrum (DMSO-d$_6$)

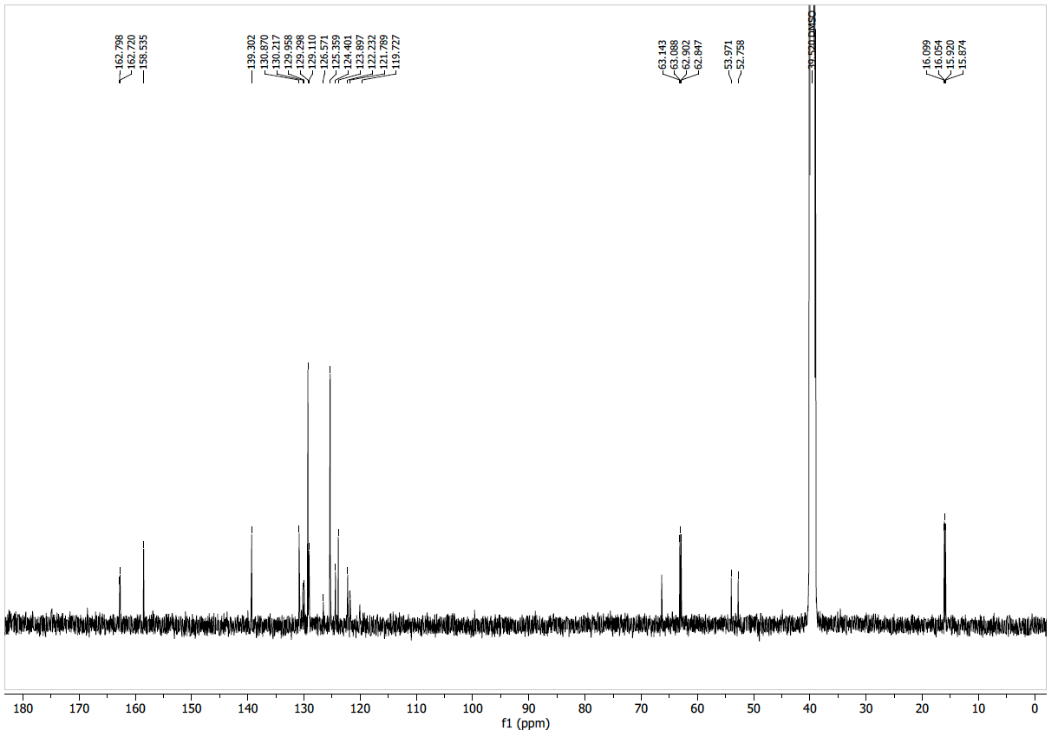

^{13}C{^{1}H} NMR spectrum (DMSO-d$_6$)

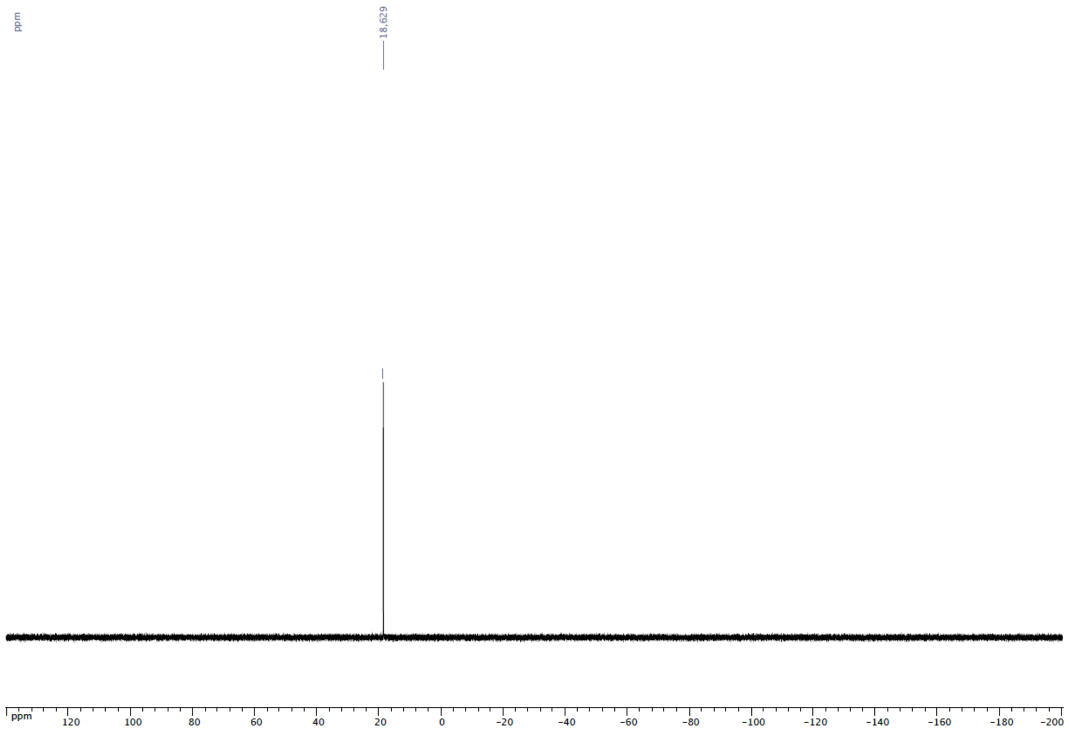

$^{31}P\{^1H\}$ NMR spectrum (DMSO-d_6)

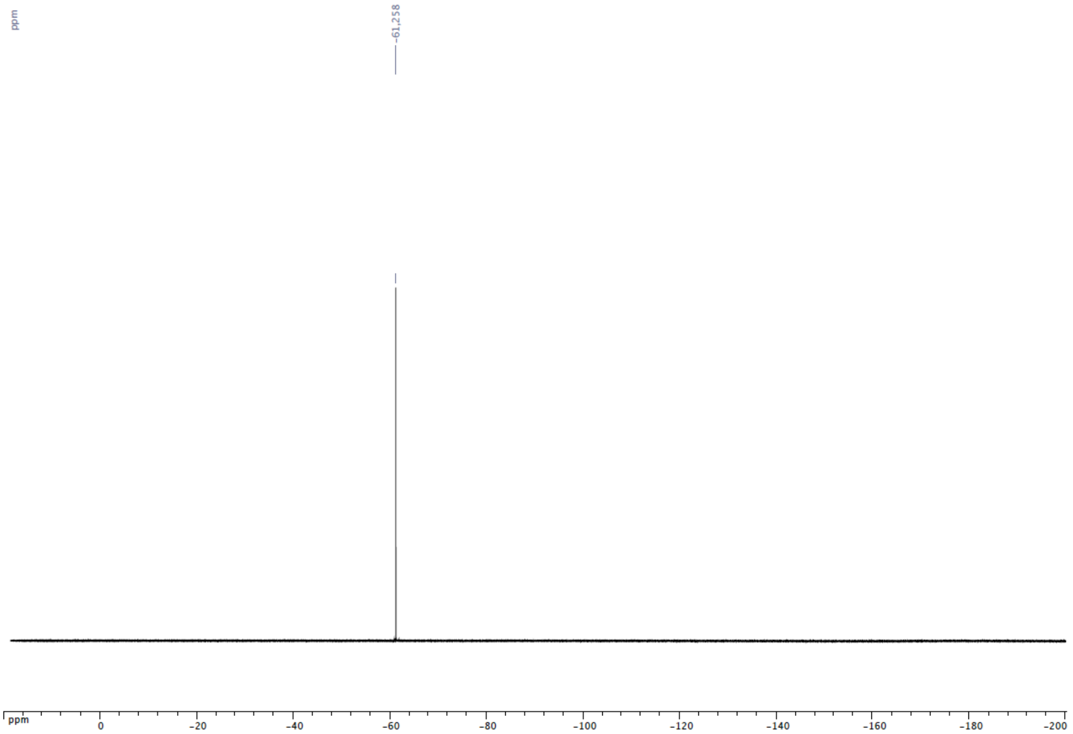

$^{19}F\{^1H\}$ NMR spectrum (DMSO-d_6)

Characterising data of diethyl[(5-phenyl-1,3,4-axodiazol-2-ylamino)(4-trifluoromethoxyphenyl)methyl]phosphonate (5f)

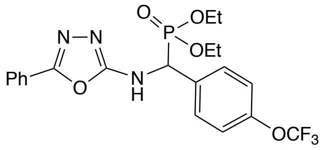

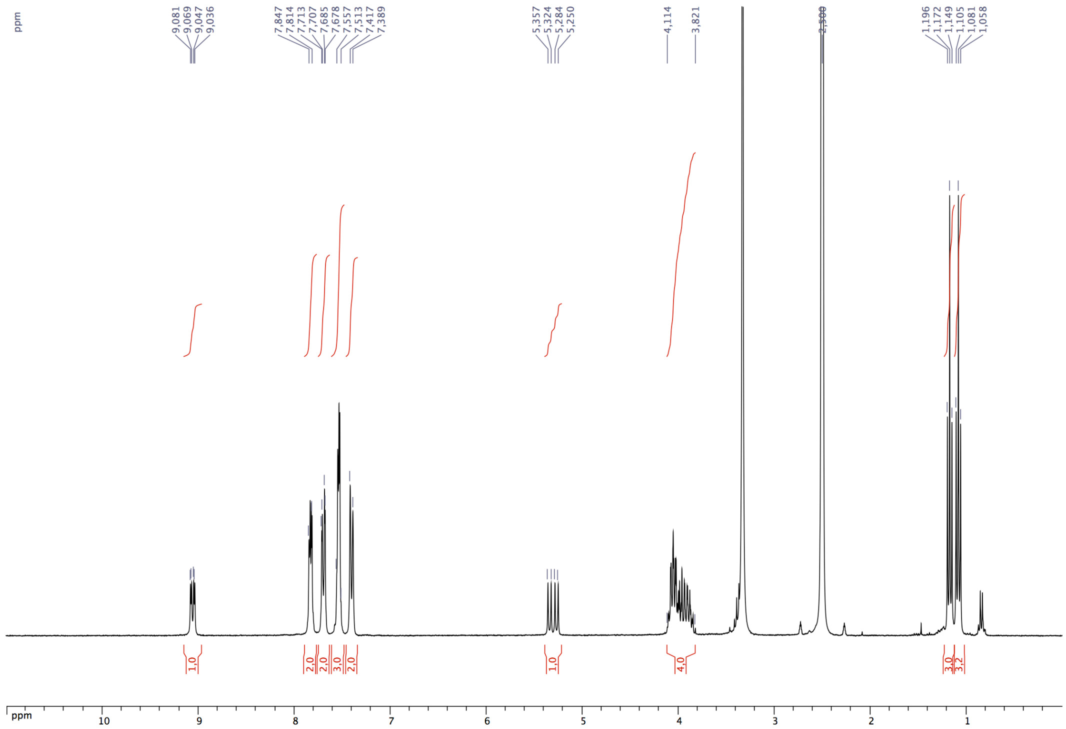

^{1}H NMR spectrum (DMSO-d$_6$)

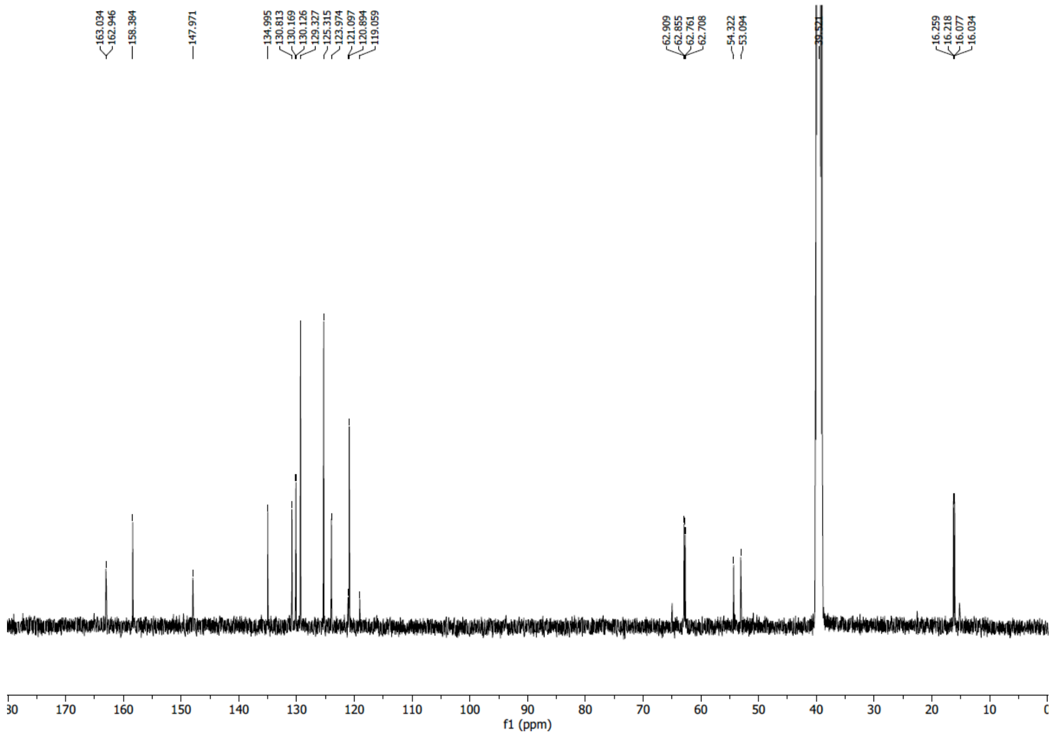

^{13}C{^{1}H} NMR spectrum (DMSO-d$_6$)

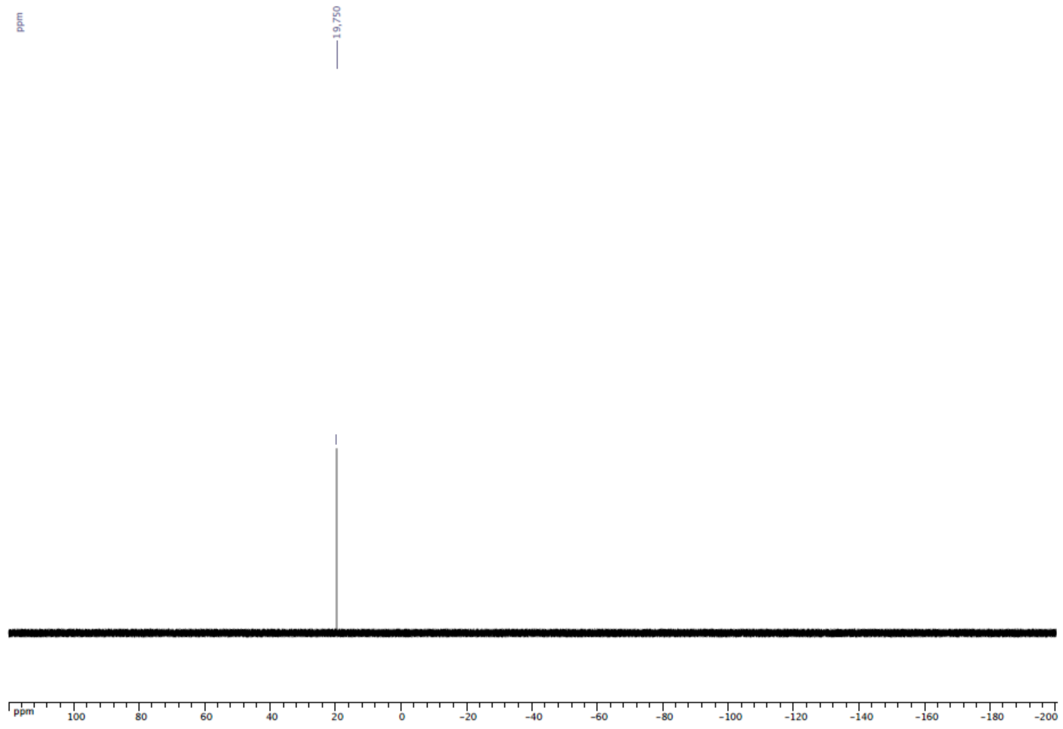

$^{31}P\{^{1}H\}$ NMR spectrum (DMSO-d$_6$)

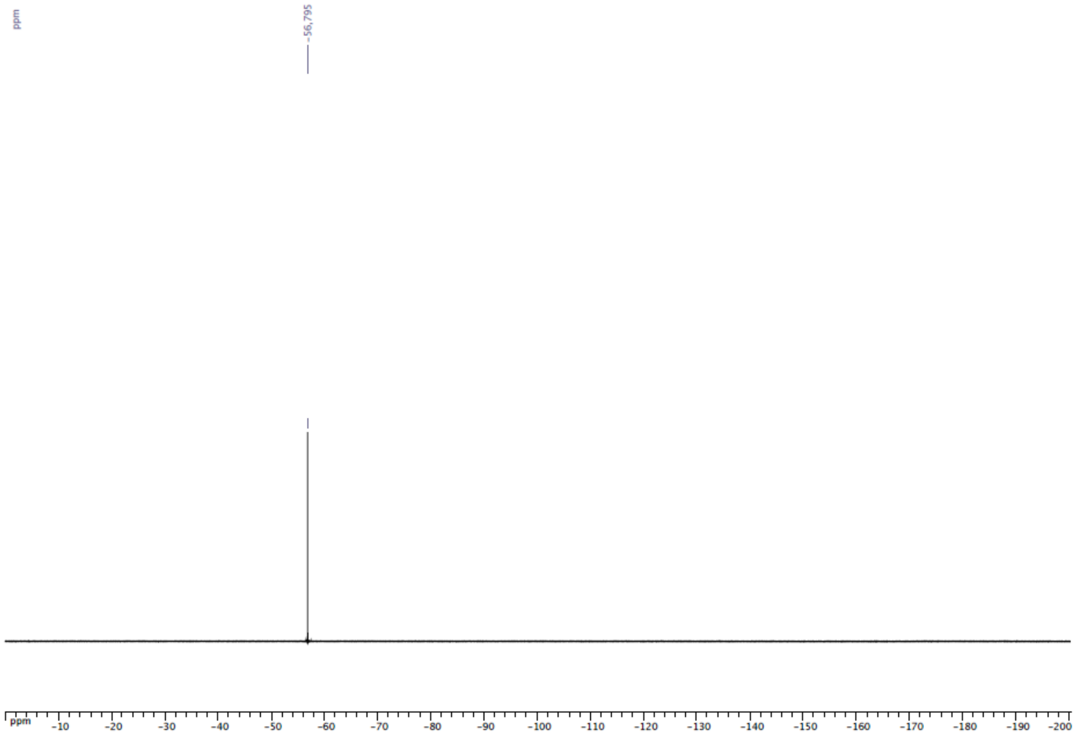

$^{19}F\{^{1}H\}$ NMR spectrum (DMSO-d$_6$)

Characterising data of [(5-phenyl-1,3,4-axodiazol-2-ylamino) (4-trifluoromethylphenyl) methyl]phosphonic acid (6a)

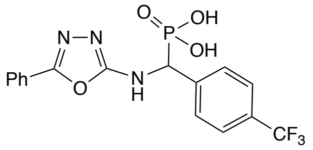

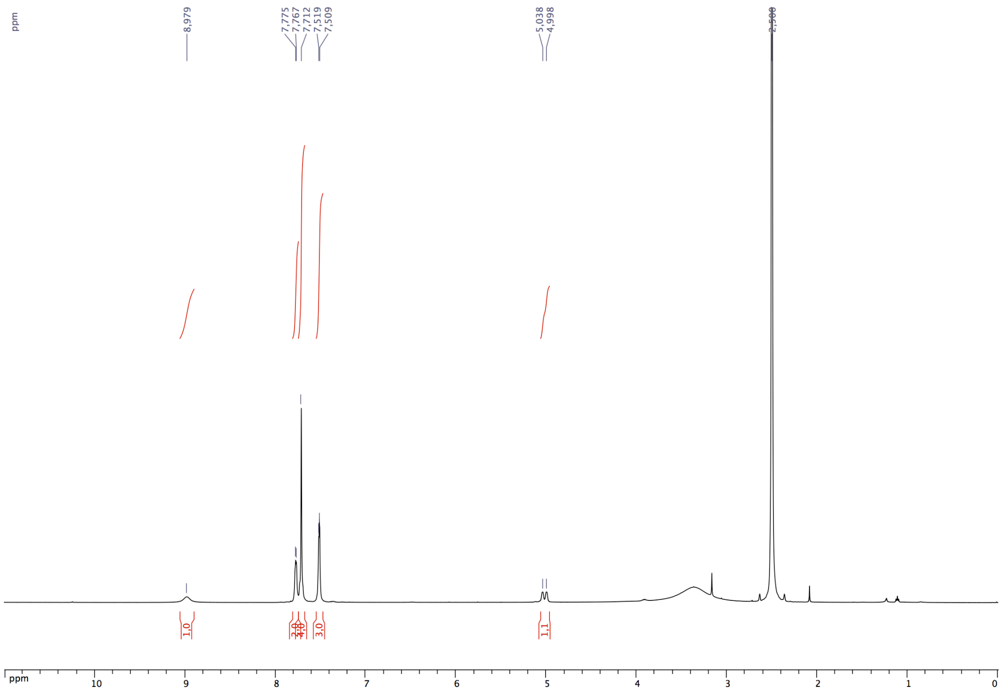

^{1}H NMR spectrum (DMSO-d_6)

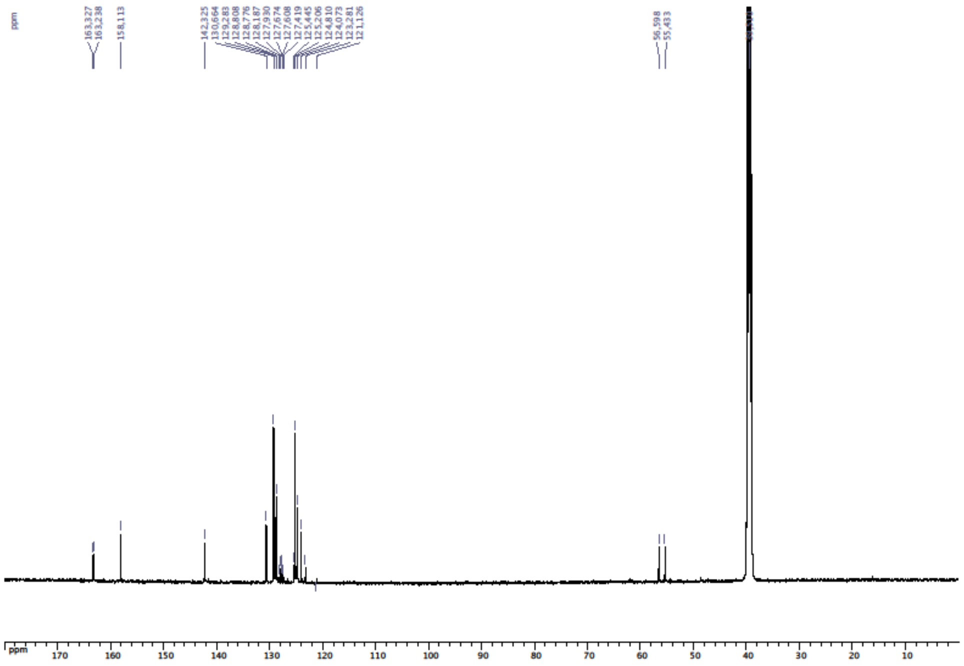

^{13}C{^{1}H} NMR spectrum (DMSO-d_6)

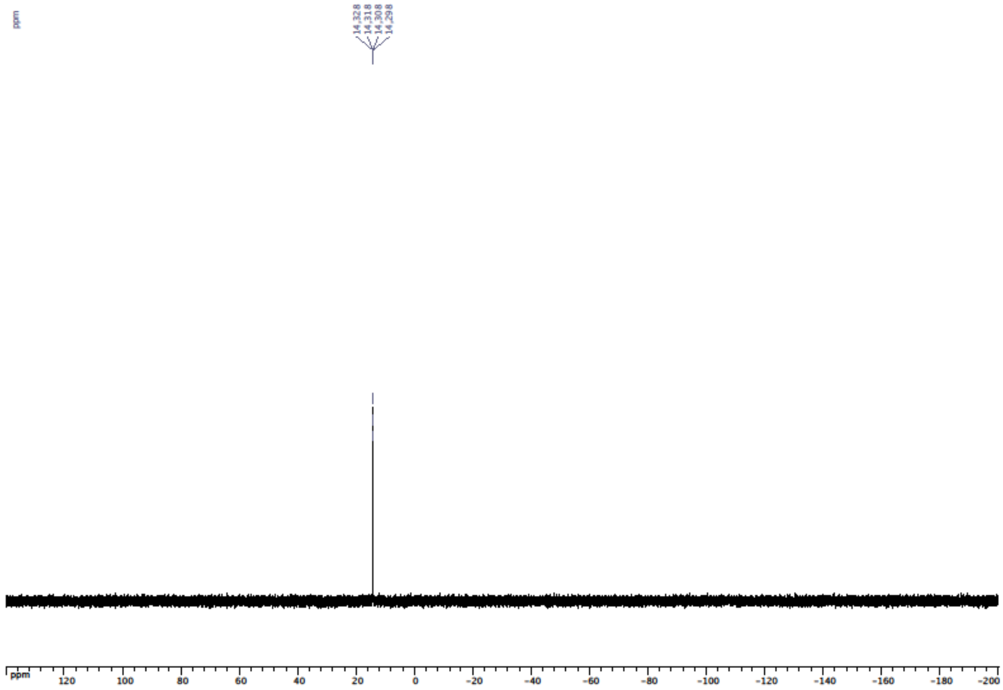

$^{31}P\{^{1}H\}$ NMR spectrum (DMSO-d_6)

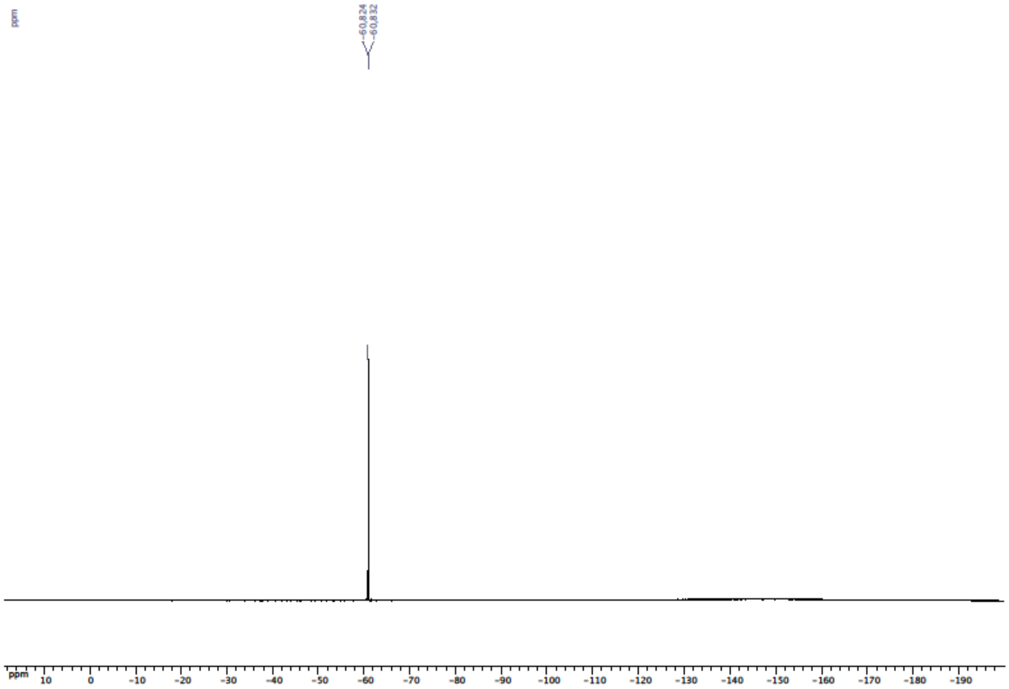

$^{19}F\{^{1}H\}$ NMR spectrum (DMSO-d_6)

Characterising data of [(5-phenyl-1,3,4-axodiazol-2-ylamino)(4-nitrophenyl)methyl]phosphonic acid (6b)

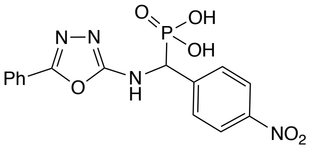

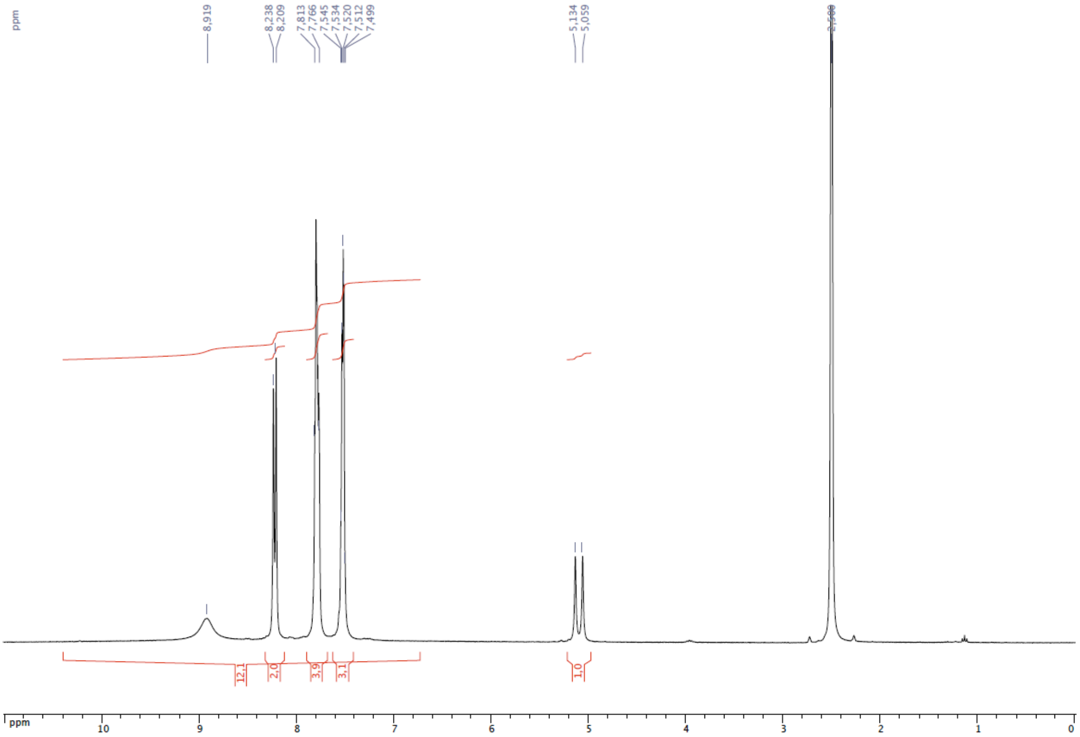

^{1}H NMR spectrum (DMSO-d_6)

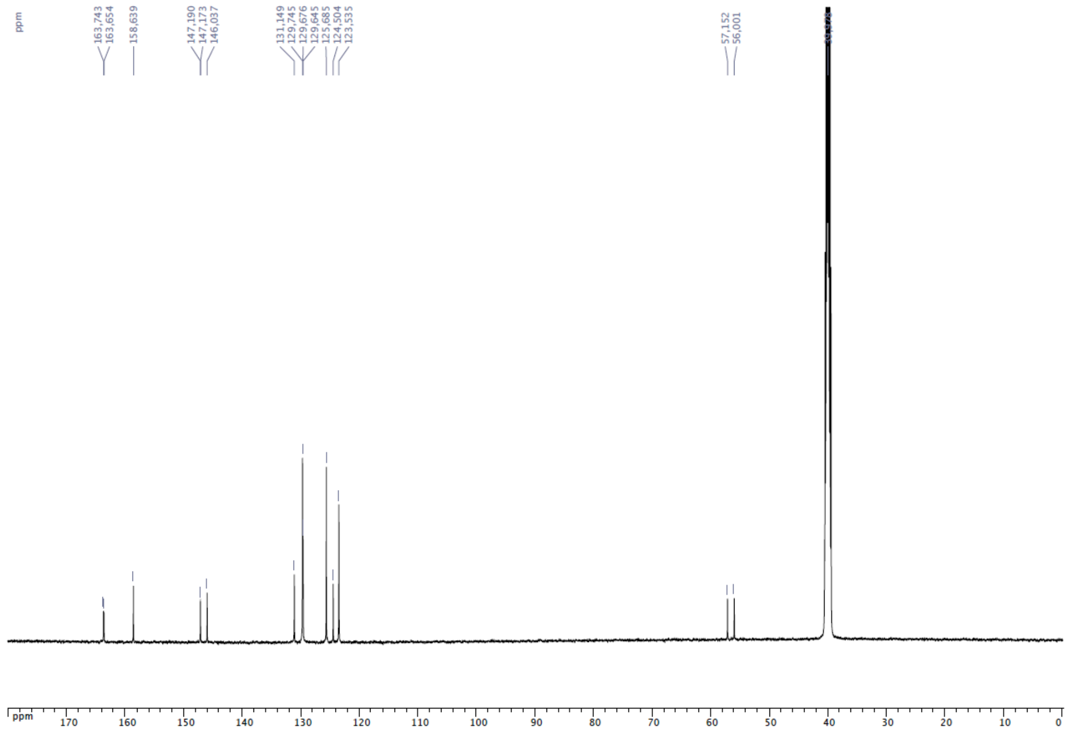

$^{13}C\{^{1}H\}$ NMR spectrum (DMSO-d_6)

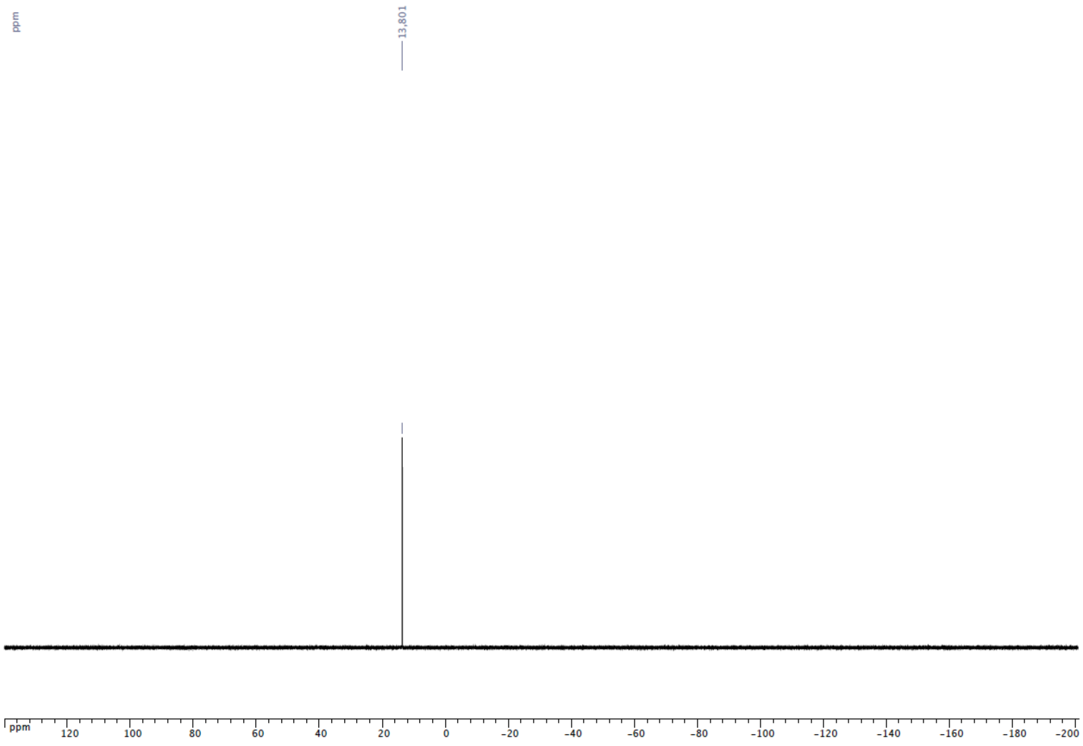

$^{31}P\{^{1}H\}$ NMR spectrum (DMSO-d_6)

**Characterising data of [(5-phenyl-1,3,4-axodiazol-2-ylamino)
(4-fluorophenyl)methyl]phosphonic acid (6c)**

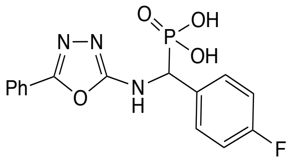

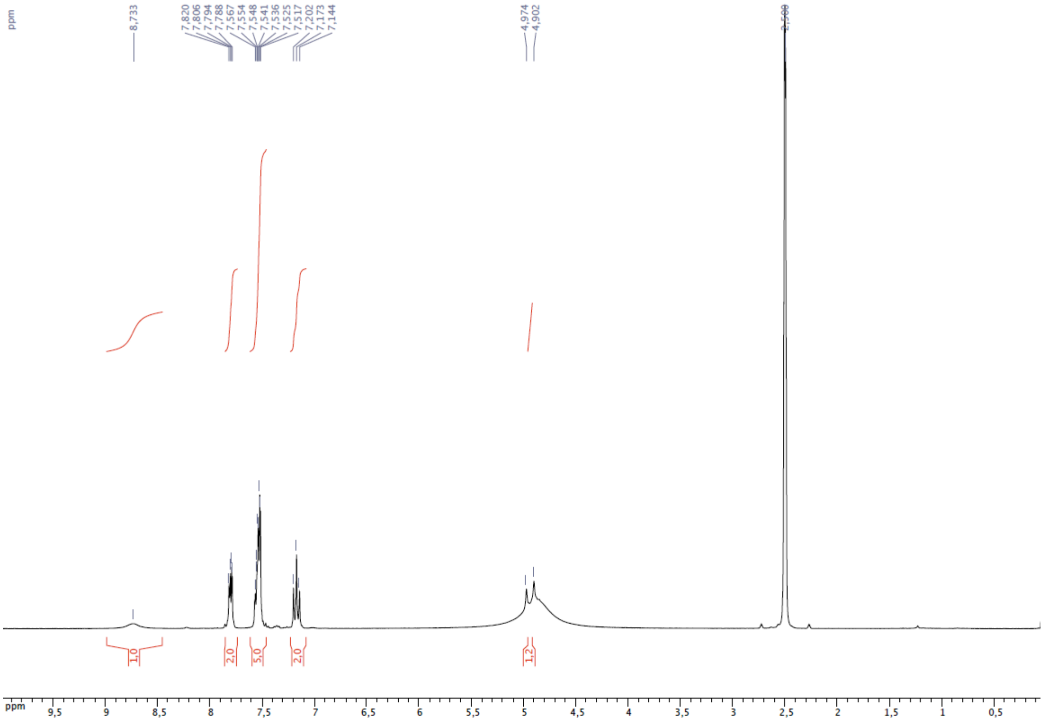

^{1}H NMR spectrum (DMSO-d$_6$)

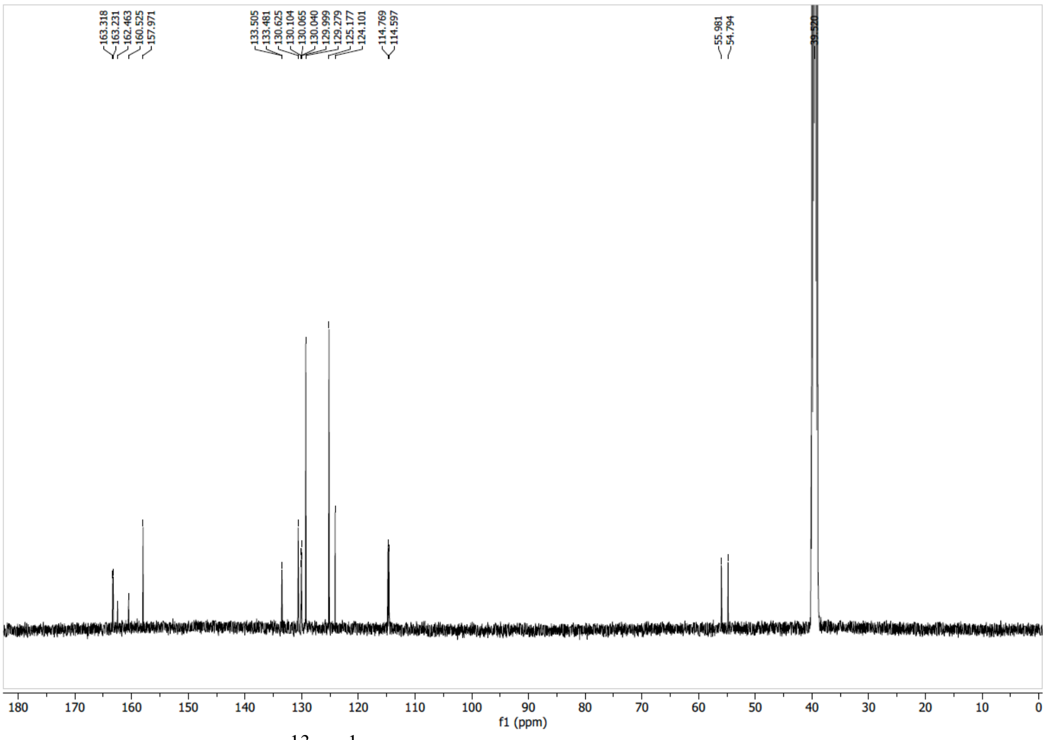

^{13}C{^{1}H} NMR spectrum (DMSO-d$_6$)

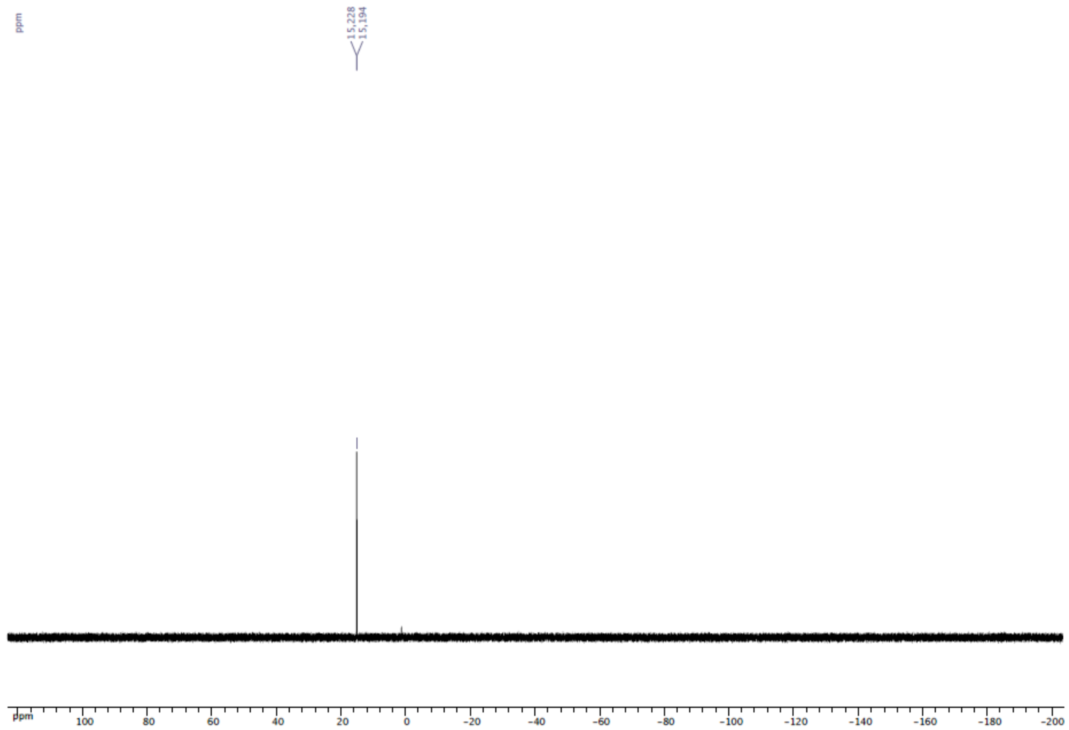

$^{31}P\{^{1}H\}$ NMR spectrum (DMSO-d_6)

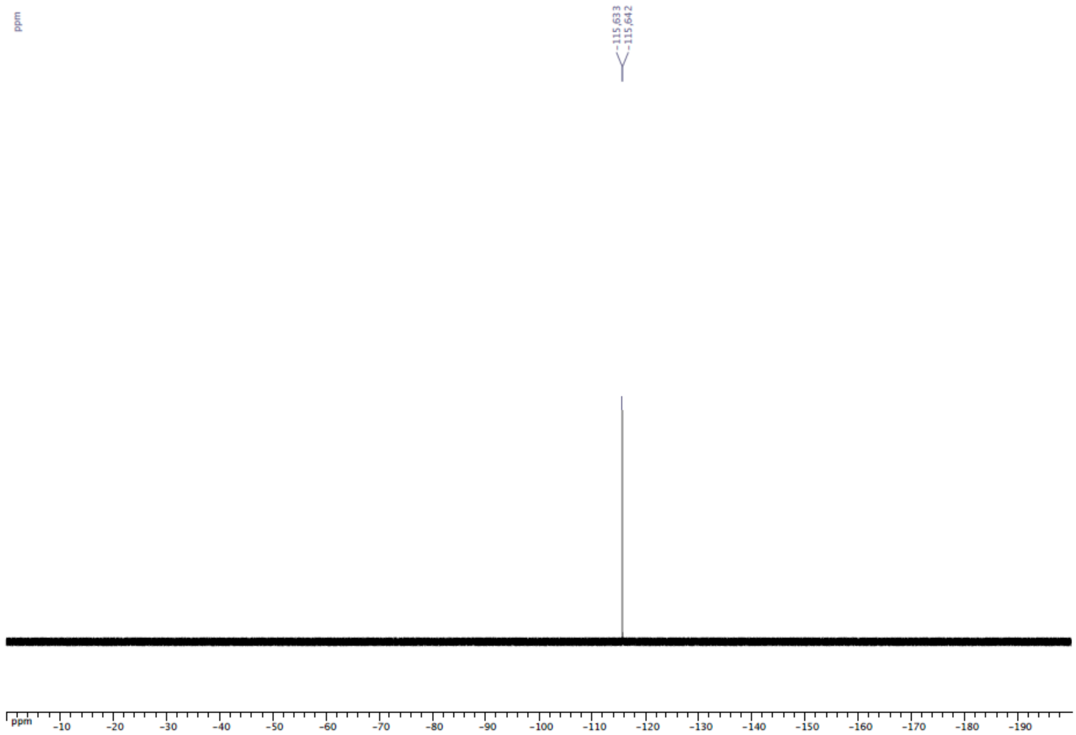

$^{19}F\{^{1}H\}$ NMR spectrum (DMSO-d_6)

**Characterising data of [(5-phenyl-1,3,4-axodiazol-2-ylamino)
(3,4,5-trifluophenyl)methyl]phosphonic acid (6d)**

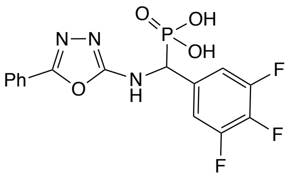

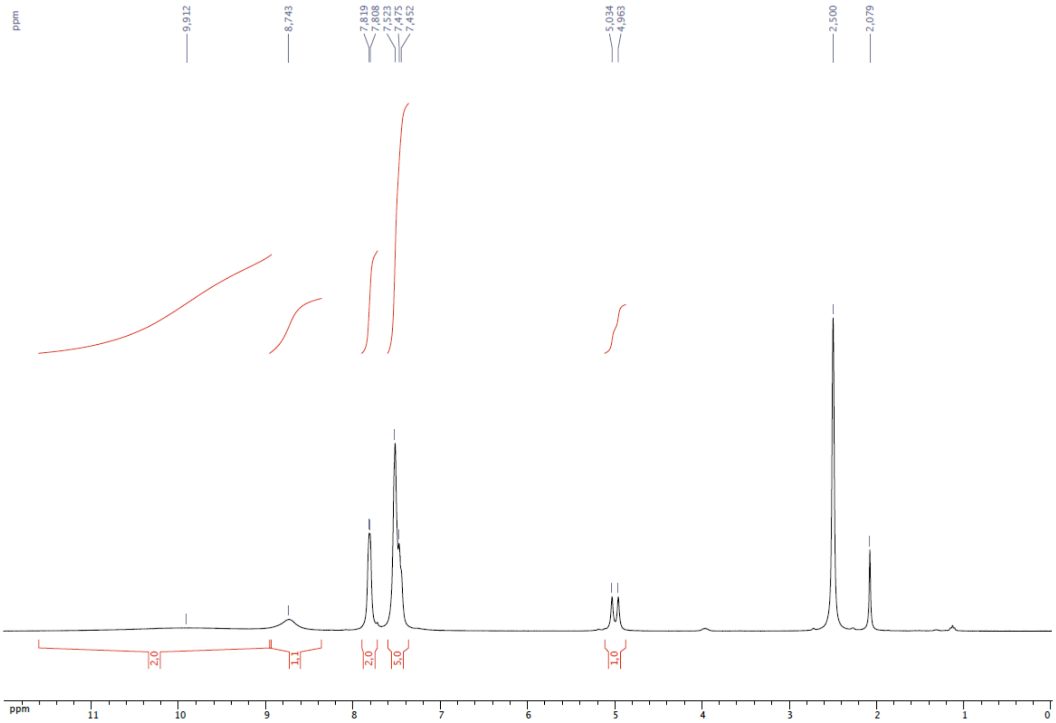

^{1}H NMR spectrum (DMSO-d$_6$)

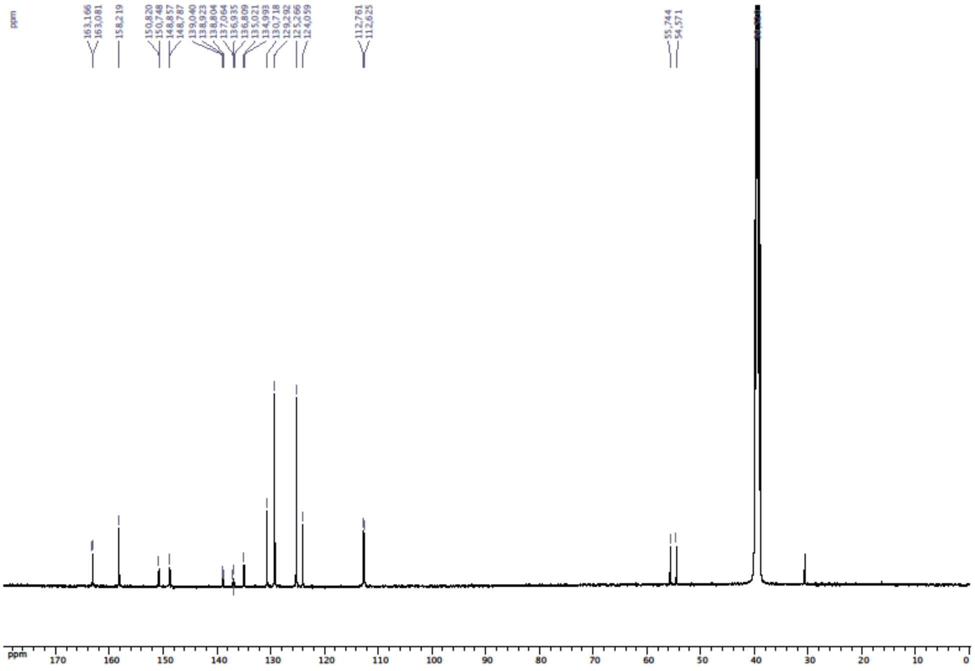

^{13}C{^{1}H} NMR spectrum (DMSO-d$_6$)

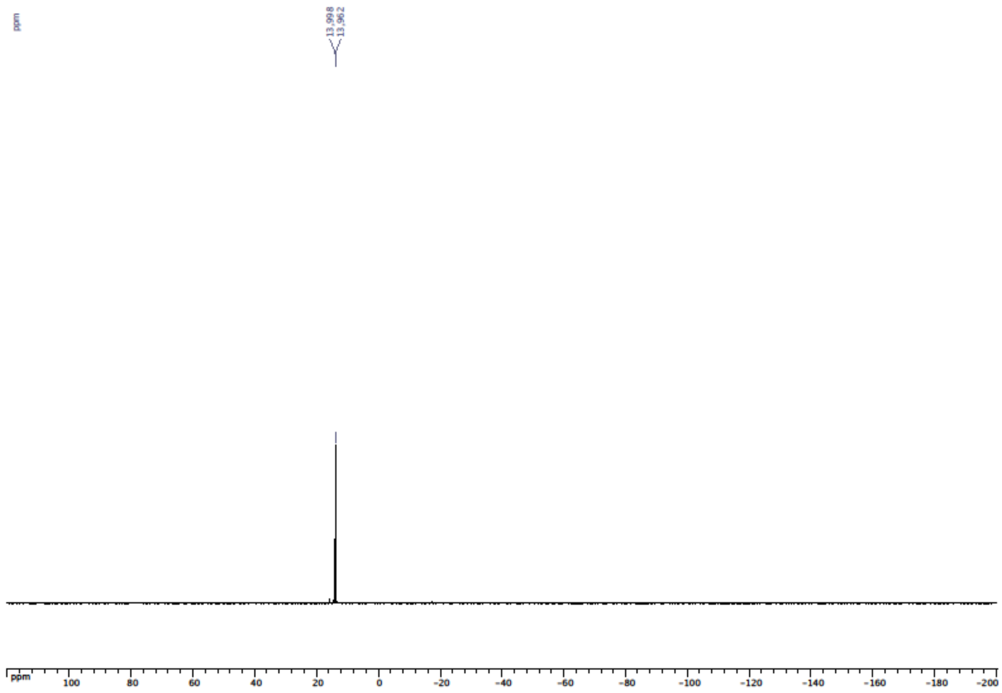

$^{31}P\{^{1}H\}$ NMR spectrum (DMSO-d_6)

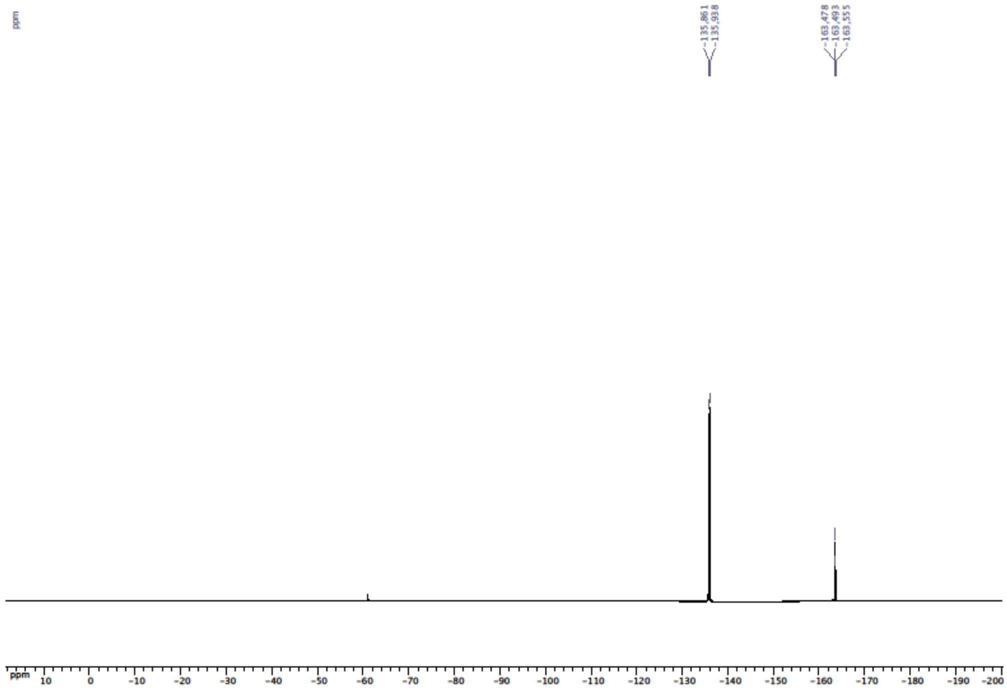

$^{19}F\{^{1}H\}$ NMR spectrum (DMSO-d_6)

Characterising data of [(5-phenyl-1,3,4-axodiazol-2-ylamino)(3,5-bis-trifuoromethyl-phenyl)methyl]phosphonic acid (6e)

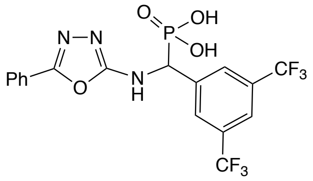

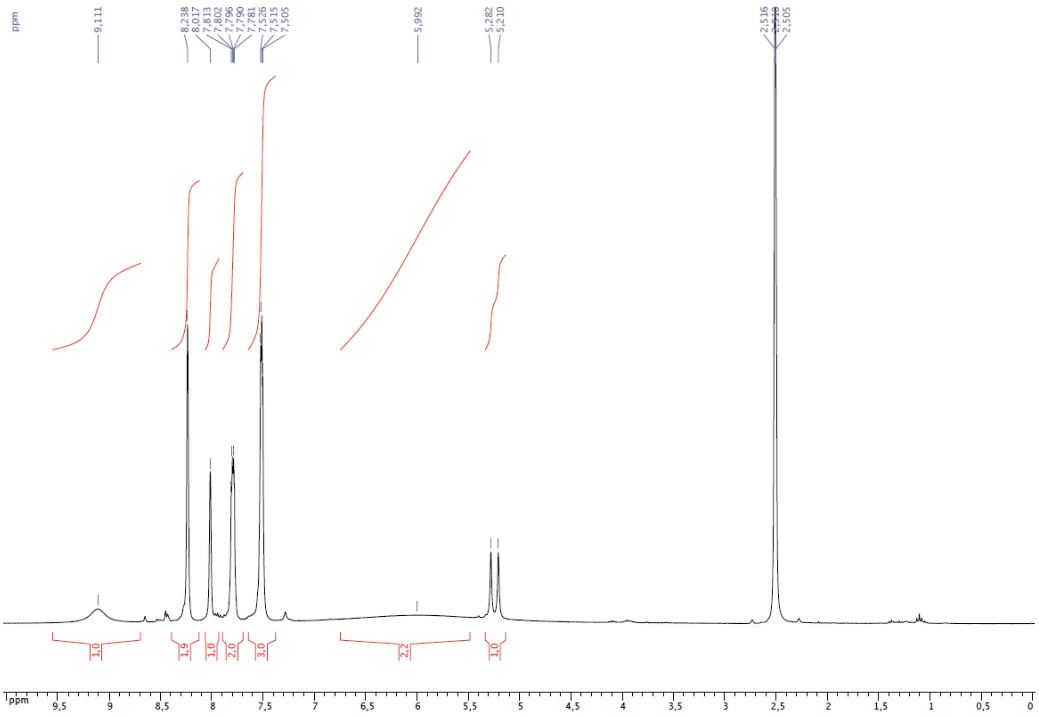

^{1}H NMR spectrum (DMSO-d_6)

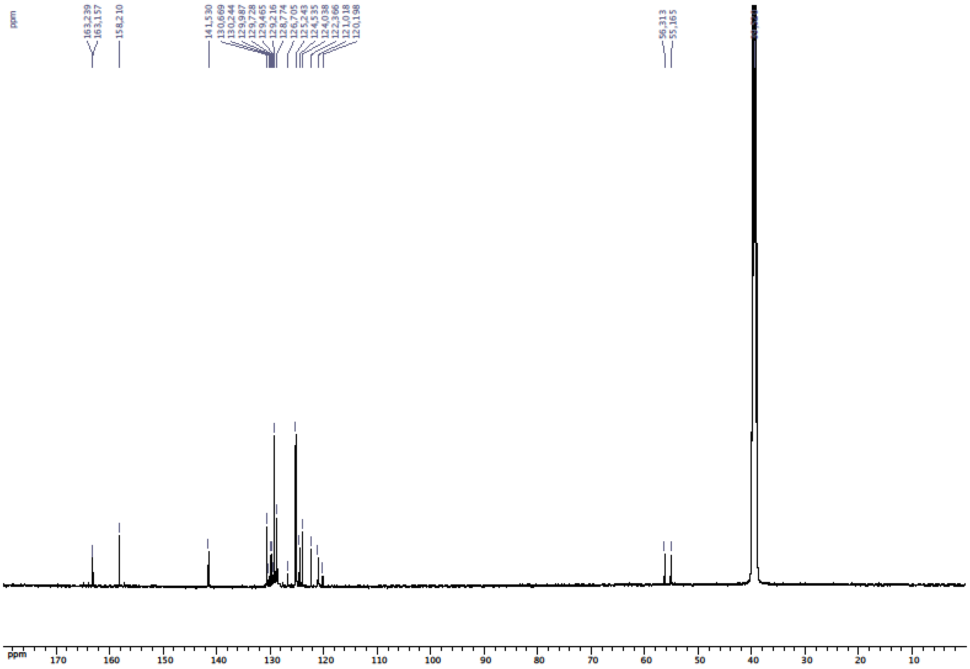

^{13}C{^{1}H} NMR spectrum (DMSO-d_6)

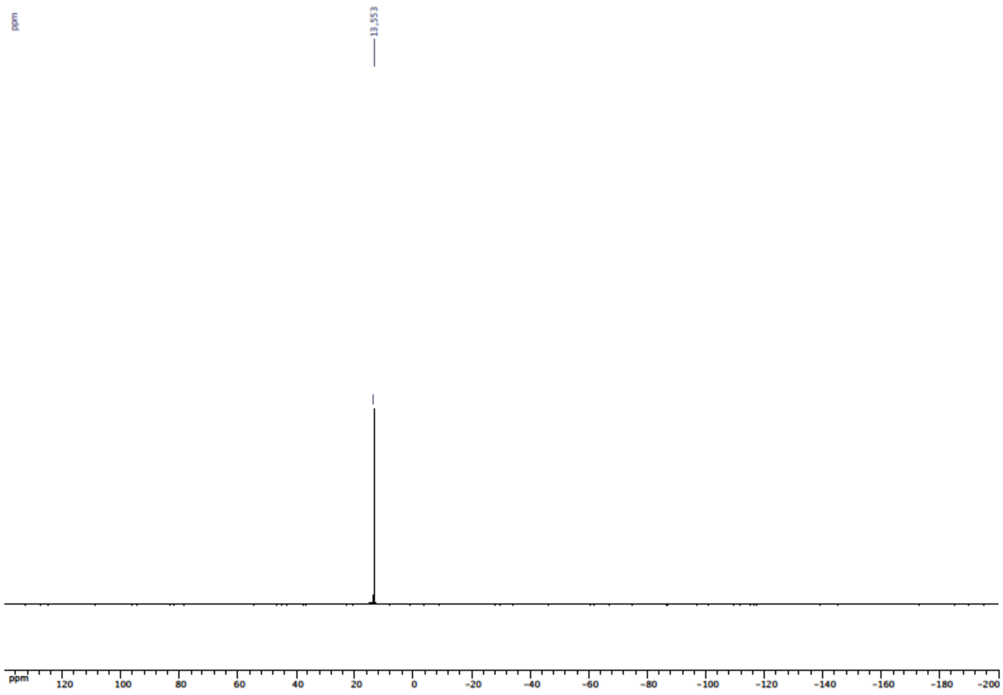

$^{31}P\{^1H\}$ NMR spectrum (DMSO-d_6)

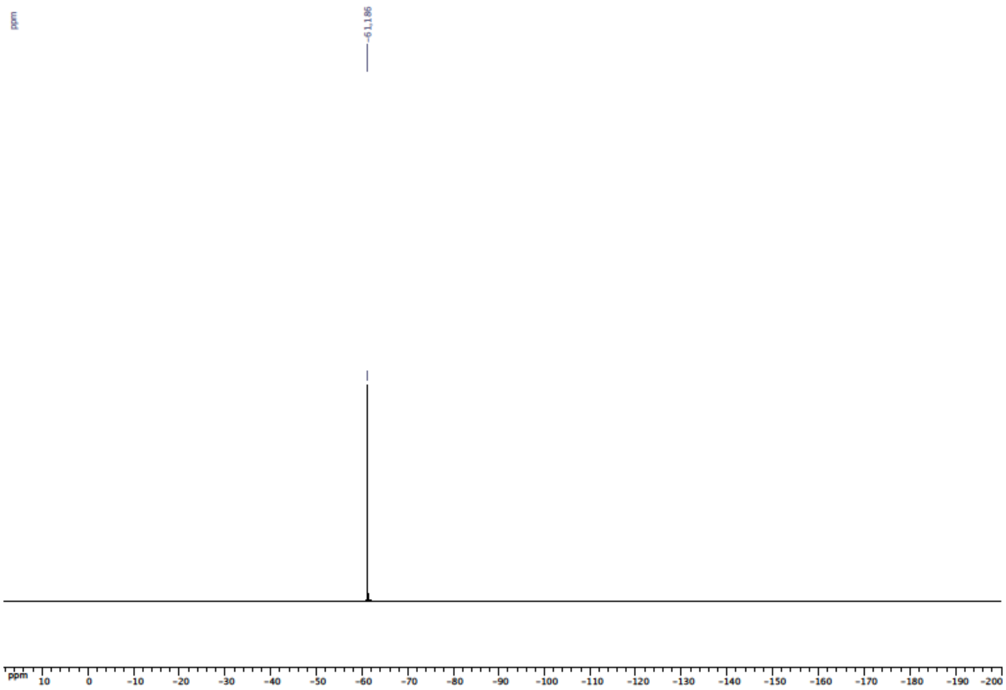

$^{19}F\{^1H\}$ NMR spectrum (DMSO-d_6)

X-ray structure of 5b

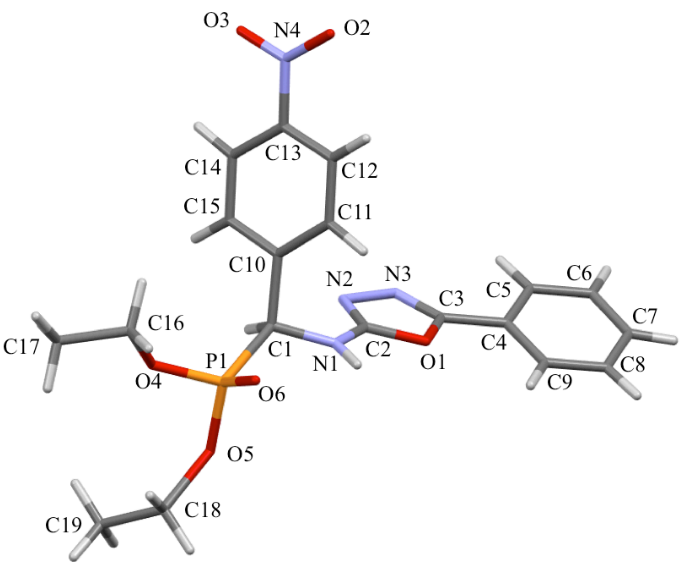

Lengths (Å)		
P1 - O6 1.458(3)	C5 - H5 0.9500	C15 - H15 0.9500
P1 - O4 1.486(3)	C6 - C7 1.303(5)	N4 - O3 1.149(5)
P1 - O5 1.547(3)	C6 - H6 0.9500	N4 - O2 1.286(5)
P1 - C1 1.980(4)	C7 - C8 1.448(6)	O4 - C16 1.346(5)
C1 - N1 1.351(4)	C7 - H7 0.9500	C16 - C17 1.486(6)
C1 - C10 1.495(4)	C8 - C9 1.353(5)	C16 - H16A 0.9900
C1 - H1 1.0000	C8 - H8 0.9500	C16 - H16B 0.9900
N1 - C2 1.385(5)	C9 - H9 0.9500	C17 - H17A 0.9800
N1 - H1A 0.8800	C10 - C15 1.307(5)	C17 - H17B 0.9800
C2 - O1 1.267(4)	C10 - C11 1.468(6)	C17 - H17C 0.9800
C2 - N2 1.286(5)	C11 - C12 1.353(4)	O5 - C18 1.635(5)
N2 - N3 1.438(4)	C11 - H11 0.9500	C18 - C19 1.540(6)
N3 - C3 1.214(4)	C12 - C13 1.293(5)	C18 - H18A 0.9900
C3 - C4 1.409(4)	C12 - H12 0.9500	C18 - H18B 0.9900
C3 - O1 1.422(4)	C13 - N4 1.419(4)	C19 - H19A 0.9800
C4 - C9 1.320(4)	C13 - C14 1.445(6)	C19 - H19B 0.9800
C4 - C5 1.472(5)	C14 - C15 1.349(5)	C19 - H19C 0.9800
C5 - C6 1.341(5)	C14 - H14 0.9500	

Angles (°)		
O6 - P1 - O4 116.80(16)	C10 - C1 - H1 108.1	N3 - C3 - C4 125.4(3)
O6 - P1 - O5 117.93(16)	P1 - C1 - H1 108.1	N3 - C3 - O1 114.9(3)
O4 - P1 - O5 92.04(16)	C1 - N1 - C2 117.2(3)	C4 - C3 - O1 119.8(3)
O6 - P1 - C1 110.53(18)	C1 - N1 - H1A 121.4	C2 - O1 - C3 103.8(3)
O4 - P1 - C1 108.73(16)	C2 - N1 - H1A 121.4	C9 - C4 - C3 114.4(3)
O5 - P1 - C1 109.34(15)	O1 - C2 - N2 109.9(3)	C9 - C4 - C5 121.9(3)
N1 - C1 - C10 101.4(3)	O1 - C2 - N1 117.5(3)	C3 - C4 - C5 123.7(3)
N1 - C1 - P1 113.4(2)	N2 - C2 - N1 132.5(3)	C6 - C5 - C4 124.6(3)
C10 - C1 - P1 117.3(2)	C2 - N2 - N3 110.4(3)	C6 - C5 - H5 117.7
N1 - C1 - H1 108.1	C3 - N3 - N2 101.0(3)	C4 - C5 - H5 117.7
C7 - C6 - C5 113.1(4)	C11 - C12 - H12 124.8	C16 - C17 - H17A 109.5
C7 - C6 - H6 123.4	C12 - C13 - N4 110.3(4)	C16 - C17 - H17B 109.5
C5 - C6 - H6 123.4	C12 - C13 - C14 125.2(3)	H17A - C17 - H17B 109.5
C6 - C7 - C8 122.8(3)	N4 - C13 - C14 124.5(3)	C16 - C17 - H17C 109.5
C6 - C7 - H7 118.6	C15 - C14 - C13 124.4(3)	H17A - C17 - H17C 109.5
C8 - C7 - H7 118.6	C15 - C14 - H14 117.8	H17B - C17 - H17C 109.5
C9 - C8 - C7 125.1(3)	C13 - C14 - H14 117.8	P1 - O5 - C18 125.8(2)
C9 - C8 - H8 117.5	C10 - C15 - C14 112.3(4)	C19 - C18 - O5 114.9(3)
C7 - C8 - H8 117.5	C10 - C15 - H15 123.9	C19 - C18 - H18A 108.5
C4 - C9 - C8 112.4(3)	C14 - C15 - H15 123.9	O5 - C18 - H18A 108.5
C4 - C9 - H9 123.8	O3 - N4 - O2 126.1(3)	C19 - C18 - H18B 108.5
C8 - C9 - H9 123.8	O3 - N4 - C13 110.1(4)	O5 - C18 - H18B 108.5
C15 - C10 - C11 122.1(3)	O2 - N4 - C13 123.8(3)	H18A - C18 - H18B 107.5
C15 - C10 - C1 110.8(3)	C16 - O4 - P1 114.8(3)	C18 - C19 - H19A 109.5
C11 - C10 - C1 127.0(3)	O4 - C16 - C17 102.1(4)	C18 - C19 - H19B 109.5
C12 - C11 - C10 125.7(3)	O4 - C16 - H16A 111.3	H19A - C19 - H19B 109.5
C12 - C11 - H11 117.1	C17 - C16 - H16A 111.3	C18 - C19 - H19C 109.5
C10 - C11 - H11 117.1	O4 - C16 - H16B 111.3	H19A - C19 - H19C 109.5
C13 - C12 - C11 110.4(4)	C17 - C16 - H16B 111.3	H19B - C19 - H19C 109.5
C13 - C12 - H12 124.8	H16A - C16 - H16B 109.2	

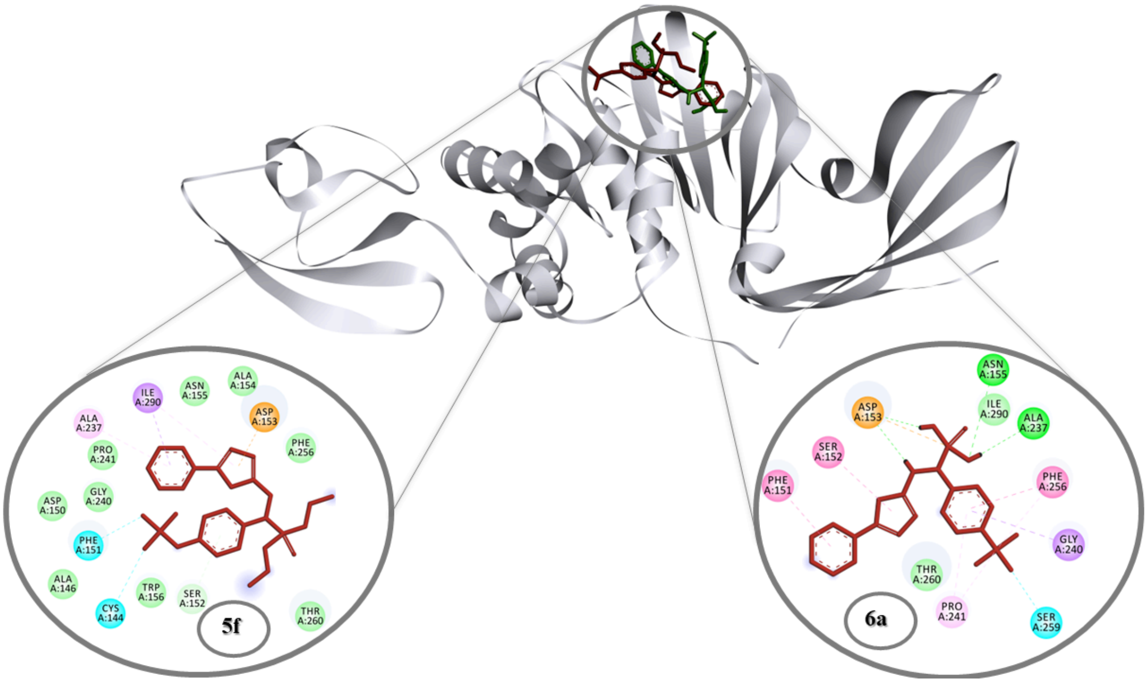

Interaction type and interaction residues of **5f** and **6a** with Papain-Like Protease (PDB ID: 4x2z) crystal structure

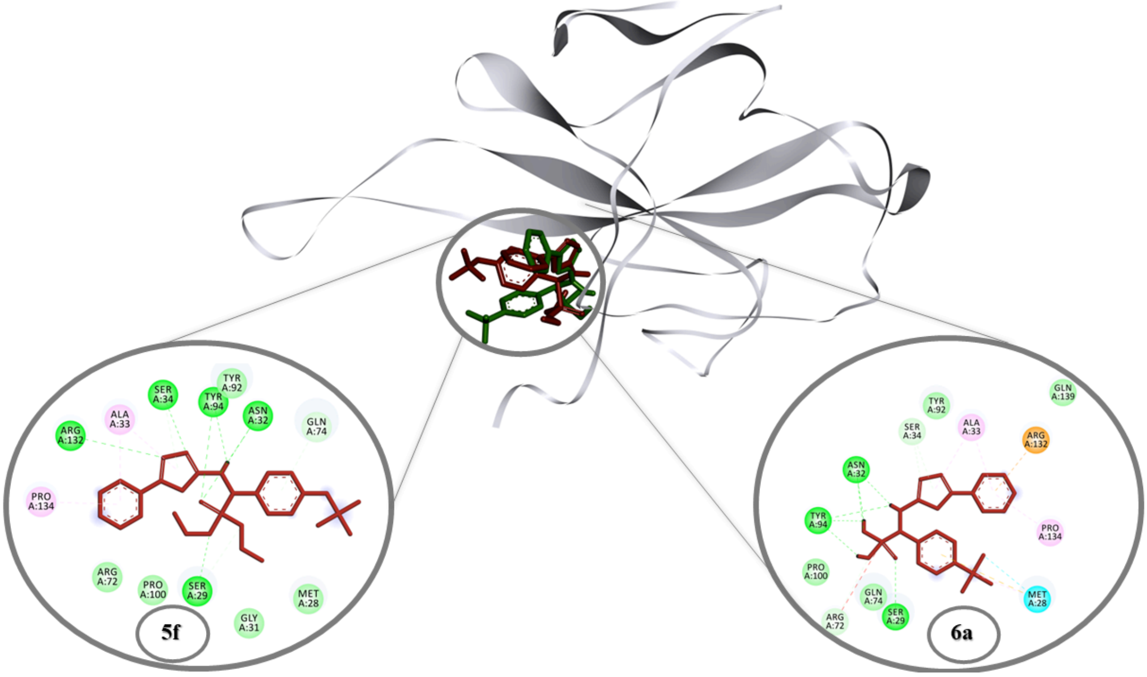

Interaction type and interaction residues of **5f** and **6a** with N- and C-terminal domain of coronavirus nucleocapsid protein (PDB ID: 2c86) crystal structure
